# Mapping and comparing the quality of life outcomes in childhood and adolescent and young adult cancer survivors: an umbrella review and future directions

**DOI:** 10.1007/s11136-024-03825-7

**Published:** 2024-12-19

**Authors:** Céline Bolliger, Kirsty Way, Gisela Michel, Samantha C. Sodergren, Anne-Sophie Darlington, On behalf of the EORTC Quality of Life Group

**Affiliations:** 1 Faculty of Health Sciences and Medicine, University of Lucerne, Lucerne, Switzerland; 2 School of Health Sciences, University of Southampton, University Road, Southampton, SO17 1BJ UK; 3 Swiss School of Public Health, Zurich, Switzerland

**Keywords:** Childhood cancer survivor, Pediatric cancer, Adolescent and young adult cancer survivors, Quality of Life, Health-related quality of life

## Abstract

**Background:**

A cancer diagnosis early in life can leave a legacy in terms of compromised Quality of Life (QoL). There is a lack of clarity regarding the impact on QoL according to age at diagnosis, with childhood cancer survivors (CCS) and adolescents and young adult cancer survivors (AYACS) often combined. As part of an EORTC Quality of Life Group study, this umbrella review aims to (1) identify the QoL outcomes reported in the literature for both CCS and AYACS, and (2) investigate the similarities and differences in QoL challenges between both groups.

**Methods:**

A systematic literature search of systematic reviews and meta-analyses was conducted in December 2023 using PubMed, PsychInfo, and CINAHL. Methodological quality was evaluated using the AMSTAR tool.

**Results:**

Overall, 1457 articles were assessed, and 39 systematic reviews and meta-analyses met the inclusion criteria. QoL outcomes were categorized into eight QoL domains, all of which were reported in both groups of young survivors. However, reviews on CCS often focused on outcomes relating to emotional functioning, cognitive difficulties, social challenges, school functioning, body image and overall happiness, whereas AYACS reviews had a greater focus on depressive symptoms, outcomes related to sexual health and reproductive health, employment, financial difficulties, self-image and identity and the impact of cancer.

**Conclusion:**

This umbrella review comprehensively explores QoL outcomes among CCS and AYACS, revealing both shared and distinct challenges. Future research should focus on developing tailored questionnaires, emphasizing transition periods and incorporating a life perspective to capture unique developmental tasks of young survivors.

**Supplementary Information:**

The online version contains supplementary material available at 10.1007/s11136-024-03825-7.

## Introduction

A cancer diagnosis early in life can have a profound impact on both physical health and overall well-being, influencing an individual’s quality of life (QoL) extending beyond the initial diagnosis and treatment. QoL serves as a critical outcome measure in clinical oncology research, encompassing various aspects of functioning (e.g., physical, emotional, social, role) and symptoms (e.g., pain, fatigue) [1, 2]. The specific impact of age at diagnosis in (1) children, and (2) adolescents and young adults (AYAs) may uncover both shared and distinct QoL challenges. Despite the low incidence of childhood cancer, survival rates exceed 80% in most European and North American countries, resulting in an increasing number of childhood cancer survivors [3]. On the other hand, the incidence of cancer in AYAs has been steadily increasing for decades, but morbidity and mortality have declined [4, 5]. Research often distinguishes between children with cancer, AYAs with cancer, and adults with cancer due to differences in tumor biology and psychosocial needs [6, 7]. *Childhood cancer survivors* (CCS) are often defined as individuals diagnosed with cancer between the ages 0–14 years [8]. However, there is a global heterogeneity in the upper age thresholds used to define childhood, sometimes including adolescents up to 21 years, which are often determined by the diversity of age groups included in the large contemporary childhood cancer cohorts [9–11]. *Adolescent and young adult cancer survivors* (AYACS) are often defined as those diagnosed between 15 and 39 years of age [12]. Inconsistencies in the definitions of CCS and AYACS, and therefore overlaps in the age ranges, result in difficulties identifying the distinct QoL challenges pertinent to each age group [13].

While cancer and its aggressive treatment are toxic irrespective of age, the effects are likely to be amplified in children and AYAs given the dynamic biological and psychosocial developmental stages young patients and survivors must navigate. In line with Erikson’s Stages of Psychosocial Development [14], infancy is a vital period for emotional development and trust amongst parental and caregiver relationships. Preschool is characterized by cognitive development, including self-regulation, self-awareness, logical thinking, and social interactions. Adolescence involves identity formation and social relationship development, whereas young adulthood sees the formation of intimate, romantic relationships and independence [14, 15]. Receiving a diagnosis and undergoing treatment during any of these stages may result in an interruption to developmental milestones, and thus impact long-term psychosocial development. For example, previous research suggests that school absences during childhood cancer may cause interruptions to foundational cognitive milestones, such as literacy or numeracy [16, 17], while absences during later school years as an AYA cancer patient may result in cognitive impairments affecting career opportunities, thus inducing financial toxicity [18, 19].

Young survivors of cancer experience difficulties across different QoL domains, including social functioning, self-perception, employability, and functional ability [20–23]. However, the needs of those surviving childhood cancer are often merged with the needs of those surviving AYA cancer [24, 25], despite the two age-groups navigating different developmental milestones. Furthermore, some studies fail to clarify which groups of young survivors are being reported [26, 27]. Most knowledge surrounding the late effects of AYACS have been derived from cohorts of CCS, potentially failing to address the concerns prominent to an adolescent or young adult negotiating life after cancer, such as challenges navigating changes in sexual functioning and family planning [28].

This lack of clarity results in challenges when tailoring the unique, age-specific psychosocial support young survivors of cancer receive following their treatment [29]. To date, there has been no work directly comparing the QoL challenges experienced by both CCS and AYACS, based on their age at diagnosis. Clearer delineation is required when assessing the needs of CCS and AYACS, to ensure it is well understood *what* outcomes are prominent at *which* age as a young cancer survivor. Younger cancer survivors are likely to live with long-lasting late effects for a significant portion of their lives, therefore it is important they receive QoL support that is best suited to support them living beyond their disease.

This umbrella review navigates the literature exploring the QoL outcomes experienced by CCS and AYACS via systematic reviews and meta-analyses, as part of an EORTC Quality of Life Group study to inform QoL outcomes for young survivors of cancer. The review aims to: (1) identify the QoL outcomes reported in the literature for both CCS and AYACS, and (2) investigate the similarities and differences between QoL challenges for both groups of young survivors. This work lays the groundwork for future research into the development of tailored QoL questionnaires, interventions, and support mechanisms for CCS and AYACS.

## Materials and methods

This umbrella review followed the Preferred Reporting Items for Systematic Reviews and Meta-Analyses (PRISMA) guidelines (supplementary information 1) [30] and was registered on PROSPERO (No. CRD42024500401). The umbrella review method, chosen for its ability to synthesize evidence from multiple systematic reviews and meta-analyses, provides a broad perspective, identifies consistencies and discrepancies across studies, and contributes to scholarly knowledge [31].

### Literature search

A systematic search of the literature was conducted using the electronic databases PubMed, PsycINFO, and CINAHL. Publications clearly defined as systematic reviews, inclusive of meta-analyses, that investigated QoL in childhood, adolescent, and young adult cancer survivors were eligible for inclusion. The search strategy had five blocks: (1) *childhood* (2) *adolescence and young adulthood* (3) *cancer* (4) *quality of life* (5) *systematic review/meta-analysis*. Search terms were defined with the support of a librarian’s expertise, and controlled vocabulary (e.g., MeSH terms) were used where necessary (Supplementary information 2). The search was conducted on December 15, 2023.

### Selection criteria

We defined the inclusion and exclusion criteria a priori, as presented in Table 1.

**Table 1:** Inclusion and exclusion criteria

PICOS	Inclusion criteria	Exclusion criteria
Population	• Children, adolescents, and young adult cancer survivors diagnosed with cancer up to 39 years of age • Proxy report if participants were under the age of 16 years at the time of study	• Age at diagnosis was not available, or only age at study participation was provided
Intervention/Exposure	• Cancer survivors^a^ who were post-treatment and had no evidence of active disease	• Studies that did not identify if survivors were on or off treatment
Comparison	–	–
Outcome	• Outcomes of QoL, or HRQoL	• Palliative care • Measurement tool for QoL/HRQoL
Study design	• Systematic review and/or meta-analysis • Written in English • Any year of publication	• Guidelines or protocols of systematic reviews

*QoL* Quality of life; *HRQoL* Health-related quality of life

^a^For the purposes of this review, a “survivor” is defined as a cancer patient no longer receiving treatment, or who has no sign of active disease

### Study screening

All searches were performed sequentially, results were entered into a Zotero bibliography (https://zotero.org/) and duplicates were removed manually. Study titles and abstracts were independently screened by two reviewers (C.B. and S.S.) using a non-automated web tool, Rayyan (https://rayyan.ai/). Full-text screening was conducted on eligible studies, to confirm they met the inclusion criteria. Any disagreements regarding eligible studies were resolved via consensus, or by a third reviewer (K.W).

### Data extraction and analysis

Following full-text screening, two reviewers (C.B. and K.W.) completed data extraction using predefined extraction sheets. Data collection included author, year of publication, review methodology, databases searched, number of studies included in the reviews, study objectives, conclusions, population demographics (age at diagnosis and study) and the main QoL outcomes. Each review and its findings were categorized as CCS, AYACS, mixed (the sample included both CCS and AYACS), or unclear (age at diagnosis was not clear), as per the author’s classification. All QoL outcomes extracted from studies with off-treatment samples were narratively categorized [32] into a framework adapted from the work by van Leeuwen and colleagues [33], covering three key aspects of QoL: physical, mental, and social well-being. The EORTC Quality of Life Questionnaire (QLQ-C30) was used as part of this framework and included five functional scales (physical, role, cognitive, emotional, and social) and three symptom scales (fatigue, pain, and nausea/vomiting) [34]. The categorization of all QoL outcomes were critically appraised by two reviewers (C.B. and K.W.), leading to the establishment of eight domains of QoL. A meta-analysis was not performed because certain studies might have been included in more than one systematic review, which would increase the risk of bias in a meta-analysis [35].

### Quality assessment

To assess the methodological quality of the included studies (i.e., systematic literature reviews and meta-analyses; hereafter referred to as *reviews*), we used the Assessment of Multiple Systematic Reviews (AMSTAR) measurement tool [36]. This appraisal tool uses 11 questions (Supplementary information 3), each of which should be answered as “yes”, “no”, “can’t answer” or “unclear” [36]. The included reviews were classified as poor quality (AMSTAR scores ranging from 0 to 4) moderate quality (5–7) and high quality (8–11). All included reviews were scored independently by two reviewers (C.B. and K.W.) and discrepancies in scoring were resolved via an average (mean) of the two raters.

## Results

### Study selection

**Fig. 1:**
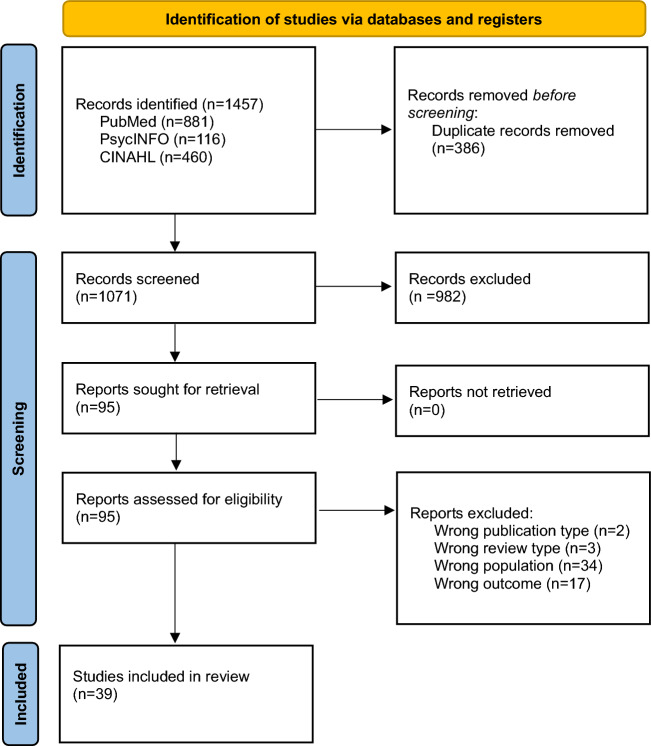
PRISMA flow diagram of included systematic reviews and meta-analyses

A total of 1457 reviews were identified from the initial literature search. After the removal of duplicates (n = 386), and title and abstract screening, a total of 95 reviews were identified for full-text analysis. Finally, 39 reviews were included in this umbrella review. The screening process is summarized in Fig. 1.

### Review characteristics

**Fig. 2:**
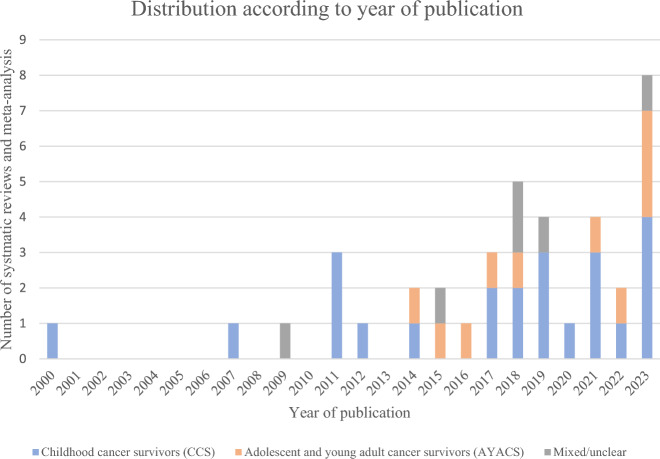
Distribution of included reviews in this umbrella and studied populations review by year of publication

All included reviews were published between 2000 and 2023, with 92% published after 2010 (Fig. 2). They referred to data from 8 to 74 original studies, that were primarily identified through the electronic databases Embase, PubMed, Medline, PsychInfo, and Cochrane. More details about the review characteristics are provided in Table 2.

**Table 2:** Characteristics and quality assessment of included reviews (n = 39)

No	Authors (Year) References	Type of review (N of studies included in this review)^a^	Databases searched	Mean AMSTAR Score^b^	Main objective	Main population^c^	Age at diagnosis in years	Age at study in years
1	Deegan et al. (2023) [38]	Systematic review (5/10)	PsychInfo, CINAHL, Embase, PubMed, Web of Science	6	Examine existing evidence on social support and outcomes such as quality of life, wellbeing and stress in child and adolescent cancer survivors	CCS	< 18	< 18
2	Larsen et al. (2023) [22]	Systematic review (36/36)	PubMed, Embase, PsychInfo, CINAHL	6	Critically evaluate and summarize the latest evidence on the QoL of CCS in Europe and to identify survivors at particular risk	CCS	15–21	15–45
3	Kappelmann et al. (2023) [39]	Systematic review (12/13)	PubMed, SPORTDiscus, Cochrane, FIS Education, Web of Science	7	Reveal the social, personal, and contextual factors that influence physical activity in children and adolescents during and after cancer treatment	CCS	< 21	< 29
4	Sciancalepore et al. (2023) [40]	Systematic review (39/39)	PubMed, Cochrane, PsycInfo, CINAHL	7	Characterize cognitive deficits and analyze their frequency in survivors of paediatric CNS tumours, at least 2 years from the end of therapies	CCS	≤ 21	NR
5	Moascato et al. (2022) [41]	Systematic review (3/14)	PubMed and PsychInfo	6	Identify predictors of adaptive family functioning, and characterize associations between family factors and the survivor’s psychosocial functioning	CCS	< 18	NR
6	Martinez-Santos et al. (2021) [50]	Mixed methods systematic review (17/21)	CINAHL, PsycInfo, Embase, MEDLINE, ERIC, Web of Science	7	Critically review and synthesize empirical studies on childhood cancer survivors’ experiences and needs on returning to school after treatment	CCS	0–18	NR
7	Pahl et al. (2021) [59]	Systematic review (36/43)	PubMed, Embase, PsycInfo	6	Examine social isolation and connectiveness in adolescents with cancer and adolescent survivors of childhood cancer	CCS	2–13	10–21
8	Schulte et al. (2021) [42]	Systematic review (73/73)	PubMed, PsycInfo, Embase, Web of Science	7	Identify the prevalence of pain in long-term survivors of childhood cancer	CCS	0–21	NR
9	Godoy et al. (2020) [43]	Systematic review (26/26)	MEDLINE/PubMed, PsychInfo, Web of Science, LILACS, IBECS	7	Investigate the effects of ALL treatment specifically on executive function and the instruments used to assess executive skills in ALL survivors	CCS	0–18	NR
10	Frederiksen et al. (2019) [37]	Systematic review (9/52)	MEDLINE/PubMed, Embase, PsycInfo	8	Evaluate and summarise the evidence on the socioeconomic conditions of childhood cancer survivors and to identify	CCS	< 20	NR
11	Garas et al. (2019) [44]	Systematic review (14/14)	Embase, MEDLINE, PsychInfo, PubMed, Cochrane	7	Identify and synthesize research on HRQoL for patients up to five years post-treatment	CCS	< 18	4–18.6
12	Nicklin et al. (2019) [51]	Systematic review (56/56)	MEDLINE, Embase, PsycInfo, PubMed, CINAHL, Cochrane	6	Identify the issues and supportive care needs of AYA survivors of a brain tumour diagnosed in childhood	CCS	Childhood brain tumour survivors	14–39
13	Vetsch et al. (2018) [45]	Systematic review (17/31)	PubMed/MEDLINE, PsycInfo, Embase, Cochrane	7	Identify ALL survivors at risk of poor HRQL and identify possible risk factors	CCS	< 18	7–54
14	Turner et al. (2018) [46]	Systematic review and meta-analysis (18/18)	MEDLINE, Embase, PILOTS, PsycInfo, Web of Science	9	Identify the demographic, medical, and psychosocial correlates of perceived post-traumatic growth in individuals of any age who were affected by pediatric cancer	CCS	< 21	< 21
15	McDonell et al. (2017) [66]	Systematic review (24/24)	MEDLINE, EMBASE, Cochrane, Web of Science, PsycInfo	6	Synthesize current knowledge about anxiety among adolescent survivors of pediatric cancer and highlights areas for future research	CCS	Pediatric cancer survivors	10–22
16	Schulte et al. (2017) [47]	Systematic review and meta-analysis (74/74)	EMBASE, PsychInfo, MEDLINE	9	Summarize studies describing HRQoL in pediatric CNS tumor survivors and compare HRQL outcomes in studies that included a comparison group	CCS	0–21	NR
17	Macartney et al. (2014) [60]	Systematic review (16/16)	CINAHL, Medline, Embase, PsycInfo	5	Describe HRQoL outcomes in pediatric brain tumor survivors, identify instruments used to measure HRQoL, and determine the relationship between symptoms and HRQoL	CCS	0.5– 6	1.5– 20
18	Pini et al. (2012) [48]	Systematic review (17/22)	MEDLINE, Embase, PsycInfo, CINAHL, ASSIA	5	Identify the impact of a cancer diagnosis on the educational engagement and school life of teenagers	CCS	13–19	NR
19	Klassen et al. (2011) [52]	Systematic review (44/58)	MEDLINE, CINAHL, Embase, PsycInfo, Cancerlit, Sociological Abstracts	5	Identify all published studies where a generic or disease specific QoL measure was used to measure child health outcomes and at least one variable in relation to QoL	CCS	Children with cancer or CCS	≤ 30
20	Nightingale et al. (2011) [53]	Systematic review of qualitative studies (16/16)	PsychINFO, PubMed, and EBSCOhost	4	Identify the domains of HRQoL that are unique to YASCC and compare these to the City of Hope Framework	CCS	Childhood cancer	13–38
21	Lund et al. (2011) [49]	Systematic review (41/41)	PubMed, Embase, PsychInfo	4	Examine the impact of childhood cancer on psychological and social outcomes in long-term survivors	CCS	< 21	Long term survivors
22	Clarke et al. (2007) [67]	Systematic review (23/23)	MEDLINE, CINHAHL, Embase, PubMed	5	Review prevalence and predictors of risk behaviors especially smoking and values of interventions to reduce risk behaviors in childhood cancer survivors	CCS	CCS, ≥ 10	> 18
23	Eiser et al. (2000) [61]	Systematic review (7/20)	PubMed, Scopus, PsychInfo	4	Determine the psychological consequences of surviving childhood cancer	CCS	CCS	3–37
24	Altherr et al. (2023) [54]	Systematic review (21/35)	PubMed, Scopus, PsychInfo	6	Describe education, employment, and financial outcomes and determinants for adverse outcomes in AYA cancer survivors	AYACS	15–39	18—≥ 81
25	Tanner et al. (2023) [55]	Systematic review (6/12)	PubMed, EMBASE, CINAHL, PsychInfo	5	Compare patient reported mental health outcomes between AYAs diagnosed with cancer and non-cancer controls	AYACS	15–39	28.5–40.2 (mean)
26	Osmani et al. (2023) [62]	Systematic review and meta- analysis (37/68)	MEDLINE/ PubMed, PsycInfo, Scopus, Web of Science	9	Examine the prevalence, risk and associated factors, and trajectories for psychological distress, anxiety, and depression among AYACS	AYACS	15–39 ± 5	15–64
27	Bradford et al. (2022) [68]	Systematic review (10/10)	MEDLINE, Embase; PsychInfo, Web of Science, CINAHL	6	Report outcomes after anti-cancer treatment for Adolescents and Young Adults	AYACS	12–29 (mean: 22)	NR
28	Stanton et al. (2018) [56]	Systematic review (11/15)	PubMed, CINAHL, PsychInfo	7	Identify the effect of cancer and its various treatment modalities on the sexual functioning of AYA men and woman	AYACS	15–39	18–40
29	Stone et al. (2017) [57]	Systematic review (8/23)	PubMed, CINAHL, PsychInfo	6	Describe what is currently known about work-related issues for young adult cancer survivors, to identify gaps in the research literature, and to suggest interventions or improvements in work processes and occupational settings	AYACS	15–39	At any time during survivorship
30	Schilstra et al. (2021) [63]	Systematic review; (15/37)	Embase, CINAHL, PsychInfo, MEDLINE	8	Synthesize available evidence – both quantitative and qualitative on how social functioning is defined in AYA psycho-oncology research and what factors are related to, and potentially predictive of, the social functioning of AYA cancer patients and survivors	AYACS	13–39 (Mean/ median)	12–46
31	Barnett et al. (2016) [58]	Systematic review (36/38)	MEDLINE/PubMed, Embase, Cochrane, Web of Science, PsycInfo	7	Identify and synthesize psychosocial outcomes, unique needs, and existing psychosocial interventions pertaining to individuals diagnosed with cancer exclusively during AYA, and to highlight areas for future research	AYACS	15–39	16–59
32	Quinn et al. (2015) [23]	Systematic review (35/35)	PubMed, PsycInfo, CINAHL	5	Identify key psychosocial factors impacting QoL in AYA oncology populations	AYACS	10–44	NR
33	Gonçalves et al. (2014) [64]	Systematic review (8/8)	PubMed, PsychInfo, CINAHL	5	Highlight what is known to date about childbearing and parenthood attitudes and decisions of young breast cancer survivors from their own perspective	AYACS	22–44	30–62
34	El Alaoui-Lasmali et al. (2023) [69]	Systematic review (58/58)	MEDLINE, Web of Science, PMC, Springer, Mary ANN Liebert, Nature Publishing Group, Eleviser, Oxford Journals, Tayler & Francis, Wiley, and Wolters Kluwer	6	Provide ways to improve the clinical practice of fertility preservation for children, adolescents, and young adults with cancer	Mixed^d^	0–25	12–53
35	Logan et al. (2019) [70]	Systematic review (15/47)	MEDLINE, Embase, PsychInfo, Web of Science, SCOPUS	6	Explore the fertility-related psychological distress and the short-term and long-term psychological impact of interrupted fertility	Unclear^d^	Reproductive age (≤ 45)	Reproductive age (≤ 45)
36	Galan et al. (2018) [71]	Systematic review (14/14)	ERIC, MEDLINE, Embase, PILOTS, ProQuest, PsycARTICLES, PsycBOOKS, psycCRITIQUES, PsycInfo, Social Services Abstracts, Sociologial Abstracts	8	Report the needs of adolescent and young adult cancer survivors after treatment	Unclear^d^	NR	14–39
37	Ismail et al. (2018) [72]	Systematic reviews (16/16)	Science Direct, PubMed and SCOPUS	5	Investigate the support needs for adolescents’ post-cancer treatment	Unclear^d^	NR	12–26
38	Olson et al. (2015) [73]	Systematic review (7/30)	MEDLINE, PubMed, Cochrane	4	Examine self-reported pain among adolescents diagnosed with leukemia or a brain tumor	Unclear^d^	NR	10–19
39	Fan et al. (2009) [74]	Systematic review (24/22)	BNI, CINAHL, MEDLINE, PsychInfo, PubMed	4	Identify differences in BI between children and adolescents with cancer and healthy controls, siblings, or other disease groups and to determine the relationships between BI and demographic or medical variables and the implications of BI for psychological adjustment and relationship between BI and social support	Mixed^d^	< 12; 13–19; >20	NR

*ALL* Acute Lymphoid Leukemia; *ASSIA* Applied Social Sciences Index and Abstracts; *AYA* Adolescent and young adult; *AYACS* Adolescent and young adult cancer survivor; *BI* Body Image; *BNI* British Nursing Index; *Cancerlit* Cancer Literature; *CCS* Childhood cancer survivor; *CNS* Central nervous system; *Cochrane* The Cochrane Collaboration; *CINAHL* Cumulative Index to Nursing and Allied Health Literature; *EBSCOhost* Electronic Business Services Corporation Host; *Embase* Excerpta Medica Database; *ERIC* Education Resources Information Center; *FIS* FIS Education electronic database; *HRQoL* Health-related quality of Life; *IBECS* Spanish Bibliographic Index of Health Sciences; *LILACS* Latin American and Caribbean Health Sciences Literature; *MEDLINE* Medical Literature Analysis and Retrieval System Online; *N* Number; *PILOTS* Published International Literature on Traumatic Stress; *PM* PubMed Central; *QoL* Quality of Life; *YASCC* Young adult survivors of childhood cancer

^a^Total number of the studies eligible for this review (i.e. patient sample were off-treatment)/total number of studies in the systematic review or meta-analysis

^b^AMSTAR Scores: mean average of two raters

^c^Main population compromises the study population at diagnosis reported by the researchers

^d^If there was no clear information about the age at diagnosis (but it was clearly not adult cancer) or study population comprises both children and AYAs, the category Unclear/mixed was used

#### Age ranges of the included reviews

A total of 23 reviews reported on CCS (59%), 10 on AYACS (26%), 4 (10%) on unclear age groups, and 2 (5%) on mixed populations (with children, and AYAs). The defined age ranges at diagnosis and study across CCS and AYACS reviews varied greatly, with limited consistency. Around 65% of the included CCS reviews defined age at diagnosis as under 18–21 years [22, 37–50], and 17% used only broader non-numeric terms like “childhood/pediatric cancer survivors” [20, 51–53]. For AYACS reviews, 50% defined survivors as aged 15–39 years [54–58]. Regarding age at study, for CCS about a third of the reviews reported specific age ranges [20, 22, 44, 45, 51, 53, 59–61], while another third did not provide any information [37, 40–43, 47, 50]. In AYACS, 70% reviews did report specific age ranges [54–56, 58, 62–64].

#### Quality of the included reviews

The quality of the included studies ranged from 3 to 9, with a mean of 6 indicating a moderate quality (see ratings of each included review in Supplementary information 4). The two main reasons for the moderate quality ratings in the studies were the lack of a comprehensive list of included and excluded studies, and the failure to clearly state potential conflicts of interest. Cohens Kappa was 0.73, indicating substantial agreement [65].

### QoL outcomes in CSS, AYACS, and unclear/mixed populations

All outcomes reported in the reviews of CCS, AYACS, and mixed/unclear populations were categorized into the following eight QoL domains (Table 3): (1) Mental health and emotional functioning, (2) Cognitive functioning, (3) Physical functioning, (4) Social and romantic relationships, sexual health, and reproductive health, (5) Health behavior, (6) Education, employment, and financial toxicity, (7) Self-perception, (8) Positive outcomes, coping, and unmet needs.

**Table 3:** Mapping QoL outcomes for CCS, AYACS, and mixed/unclear populations

	Population					
Reported outcomes	CCS (N = 23)		AYACS (N = 10)		Mixed/Unclear^a^ (N = 6)	
	References	n (%)	References	n (%)	References	n (%)
**Overall Quality of Life**^b^		8(35)		3(30)		
QoL, HRQoL	[22, 41, 44, 45, 47, 49, 51, 52]	8(35)	[23, 57, 68]	3(30)		
**1. Mental health and emotional functioning**		14(61)		9(90)		5(83)
Mental health outcomes; mental bodily changes	[45, 51]	2(9)	[55]	1(10)	[74]	1(17)
Psychological well-being; health; impact; functioning	[45, 51, 52, 61]	4(17)	[68]	1(10)	[69, 71]	2(33)
Emotional well-being; thinking; functioning; emotions	[22, 44, 45, 52, 53]	5(22)	[23]	1(10)	[70, 71]	2(33)
Depression; depressive symptoms; mood disorders	[22, 42, 45, 49, 59]	5(22)	[55, 57, 58, 62–64, 68]	7(70)	[70, 74]	2(33)
Anxiety; social anxiety; feeling scared	[22, 42, 59, 61, 66]	5(22)	[55, 58, 62, 68]	4(40)	[70]	1(17)
Suicidal risk	[22]	1(4)				
Psychotic symptoms (e.g., mood dysfunction, hallucination)	[51]	1(4)				
PTSD; PTSS; psychological stress	[38, 41, 61, 66]	4(17)	[58, 62]	2(20)		
Distress (cancer/treatment-related, psychological, emotional)	[22, 42, 45, 52]	4(17)	[57, 62, 63, 68]	4(40)	[70, 72]	2(33)
Fertility-related distress/concerns; emotional distress/worries related to infertility	[66]	1(4)			[69, 70]	2(33)
Worries (cancer/treatment-related, illness-related)	[45, 52, 66]	3(13)				
Fear of cancer recurrence/relapse; worries over future cancer	[51, 53, 66, 67]	4(17)	[23, 58, 64, 68]	4(40)	[72]	1(17)
Hostility; overwhelming; frustration; anger; shock; feelings of grief	[59]	1(4)			[70]	1(17)
Difficulty coming to terms with being neither sick nor healthy			[58]	1(10)		
Changes in personality					[74]	1(17)
Striving for normality					[72]	1(17)
Negative feelings/thoughts; feelings of losing control over life; devastation	[53]	1(4)	[58]	1(10)	[70]	1(17)
Concern/impact about life plans/future; reluctance to dream about future	[53]	1(4)			[69]	1(17)
**2. Cognitive functioning**		8(35)		2(20)		2(33)
Mental functions; cognitive (functioning, difficulties, problems, well-being); cognition; neurocognitive outcomes	[22, 45, 50, 51, 60]	5(22)	[68]	1(10)	[71]	1(17)
Intellectual functions; inhibitory control	[40, 43]	2(9)			[74]	1(17)
Executive functioning; mental flexibility; working memory; planning/organization	[40, 43]	2(9)				
Memory; short-term memory; language; attention; concentration; learning problems in school; processing speed; productivity	[40, 50, 51, 53]	4(17)	[57]	1(10)		
Visuospatial skills; spatial processing	[40, 53]	2(9)				
**3. Physical functioning**		9(39)		5(50)		4(67)
Physical (health, functioning, well-being, abilities, symptoms, issues); functional status/limitations; impact of symptoms	[22, 44, 45, 50–52, 61]	7(30)	[23, 58, 63, 68]	4(40)	[72, 74]	2(33)
Health-related disabilities; lifetime/current medical conditions; mobility	[51]	1(4)	[58]	1(10)		
Sensory/motor skills; disabilities; dexterity	[45, 50, 51, 60]	4(17)				
Fitness level	[51]	1(4)				
Role functioning; performance	[22, 45, 61]	3(13)	[54]	1(10)		
Loss of appetite; weight loss/gain	[53]	1(4)			[74]	1(17)
Fatigue; sleepiness	[42, 45, 50, 51, 53, 60]	6(26)	[23, 63]	2(20)		
Pain (e.g., before, at diagnosis, during/after treatment)	[42, 45, 60]	3(13)			[73]	1(17)
Vision; speech; hearing issues	[45, 51]	2(9)				
Discomfort; treatment discomfort	[45, 52]	2(9)				
Use of antidepressant medication			[58]	1(10)		
Vulnerability to other illnesses	[53]	1(4)				
Own health risks; perceived health			[54]	1(10)	[69]	1(17)
**4. Social and romantic relationships, sexual health and reproductve health**		15(65)		7(70)		5(83)
Psychosocial (health, consequence, impact, -concerns); social HRQoL	[48, 50, 59]	3(13)	[57, 58]	2(20)		
Social functioning	[22, 45, 49, 52, 60, 61]	6(26)				
Social (relationships, connection, adjustment, well-being, skills, experiences); communication	[41, 50, 51, 59]	4(17)	[23, 58, 63, 68]	4(40)	[71]	1(17)
Friendships (e.g., ability to maintain; awareness of others’ feelings; lack of friends; making friends); peer relationships (e.g., importance)	[49–51, 53, 59]	5(22)	[23, 68]	2(20)	[72]	1(17)
Social support from family/friends; desire to gain support from peers or peer’s survivors; importance of support; perceived social support	[38, 53, 59]	3(13)	[58]	1(10)	[72, 74]	2(33)
Social challenges (e.g., bullying and peer exclusion; teasing in school/about appearance; feeling different from peers; struggling with cancer disclosure; feelings of embarrassment; difficulties fitting in; strained relationships, less close friends; fear of negative evaluation from peers; feeling misunderstood; fear of building new relationships)	[48, 50, 51, 53, 59, 66]	6(26)	[57, 68]	2(20)		
Social isolation; loneliness; isolation from other cancer survivors/from peers without cancer; social withdrawal	[51, 59, 61]	3(13)			[71]	1(17)
Family functioning; impact on/relationship with family	[41, 44, 53, 60]	4(17)	[23]	1(10)		
Marriage outcomes	[49]	1(4)				
Psychosexual health			[58]	1(10)		
Sexual (functioning, difficulties, problems, desire, dysfunction, activity, enjoyment of activity, satisfaction); sexuality; sex drive	[22, 48, 51, 53]	4(17)	[56, 58, 68]	3(30)	[70]	1(17)
Dating					[70, 72]	2(33)
Intimate/partner relationships	[22]	1(4)	[68]	1(10)		
Fertility (issues, problems, concerns, outcomes); reproductive concerns; infertility (e.g., perception of infertility risk)	[48, 51, 66]	3(13)	[23, 58, 64, 68]	4(40)	[70, 72]	2(33)
Impact/strain of infertility on intimate relationships; risk of sterility can affect the establishment of social relationships	[53]	1(4)			[69]	1(17)
Desire to have children; family planning			[23, 58, 64]	3(30)	[69, 70]	2(33)
Concerns about passing genetic risk of cancer to future offspring; worries about cancer risk for own children; avoiding pregnancy	[66]	1(4)	[64]	1(10)	[70]	1(17)
Knowledge/opinion regarding fertility preservation; evaluation of fertility testing after cancer; impact of fertility counseling; participation in fertility preservation					[69, 70]	2(33)
Positive attitudes towards pregnancy/having children			[64]	1(10)		
**5. Health behavior**		11(48)		2(20)		2(33)
Physical activity (e.g., changes)	[38, 39]	2(9)	[58]	1(10)	[74]	1(17)
Social activities (e.g., restrictions of type of activities due to impaired skills); activities/participation; leisure time/activity (e.g., worries about missing out)	[22, 48, 50, 51]	4(17)	[58, 68]	2(20)		
Smoking (e.g. tobacco and marijuana)	[67]	1(4)	[58, 68]	2(20)		
Alcohol use; binge drinking; alcohol habits	[61, 67]	2(9)	[58, 68]	2(20)		
Drugs			[68]	1(10)		
Sleep/rest	[45]	1(4)	[58, 68]	2(20)		
Diet			[68]	1(10)		
Maintenance of healthy lifestyle; knowledge about health/disease			[58]	1(10)	[72]	1(17)
Eating disorder	[53]	1(4)				
Anti-social behavior; problem behaviors	[41, 49]	2(9)				
**6. Education, employment, and financial toxicity**		14(61)		6(60)		2(33)
Education; educational/academic achievements; educational levels; vocational levels; learning achievements	[22, 37, 48, 49, 51, 59]	6(26)	[23, 54, 68]	3(30)		
Special education needs; assistance to perform well in school; enrollment in learning disability programs; school adaption	[37, 50, 51, 60]	4(17)				
School (functioning, performance); environmental functioning; satisfaction with school environment	[44, 45, 48, 50, 60]	5(22)	[58]	1(10)	[74]	1(17)
School re-entry (e.g., new life at school, fear/concerns/anxiety, fear of failure or bullying after returning to school, anxiety about exams/ catching up on missed academic material)	[38, 48, 50, 66]	4(17)	[54]	1(10)		
School (attendance, absenteeism, status); missing school (e.g., worried about it); university attendance; sick leave	[48, 50, 51, 61]	4(17)	[54, 63, 68]	3(30)		
Academic delay; repeat a grade; disturbances in educational progress after cancer; different educational pathways	[37, 48–50]	4(17)				
Academic (life, experiences); higher degree/attending university	[37, 50, 53]	3(13)				
Work/employment (e.g., plans, changes in job due to cancer, employment after treatment; supportive workplace; reduced/perceived work ability, attending/keeping a job, status); occupational functioning; career (e.g., cancer as catalyst for change, satisfaction)	[22, 37, 48, 49, 51]	5(22)	[23, 54, 57, 58, 68]	5(50)		
Special work-related training; need support to pursue work-related goals	[51]	1(4)	[58]	1(10)		
Finances; financial (consequences, hardship; situation, difficulties)	[51]	1(4)	[23, 54, 57, 58, 68]	5(50)	[72]	1(17)
Medical financial hardships; financial declines of disability pension; impact of insurance; borrowing money			[54, 68]	2(20)		
Housing; traveling; living independently	[51]	1(4)	[68]	1(10)		
**7. Self-perception**		6(26)		5(50)		3(50)
Self-image; masculinity/femininity; self-concept	[59, 61]	2(9)	[58, 63]	2(20)	[74]	1(17)
Body (image, appearance, changes); physical appearance (e.g., hair loss, short stature, weight gain, and amputation)	[48, 51–53, 61, 66]	6(26)	[57, 58]	2(10)	[69, 72, 74]	3(50)
Self-perception; attractiveness	[61]	1(4)	[55]	1(10)	[74]	1(17)
Self-confidence; self-esteem; self-efficacy; narcissistic wounds; changing sense of self	[38, 61, 66]	3(13)			[69, 72, 74]	3(50)
Identity (cancer-related, gender-related, transformation)			[57, 68]	2(20)	[69]	1(17)
Self-care	[51]	1(4)				
Autonomy issues; sense of control over life	[60, 61]	2(9)	[57]	1(10)		
**8. Positive outcomes, coping and needs**		9(39)		5(50)		3(50)
Life (-plans,—value of importance,—enjoyment,—appreciation)	[48]	1(4)			[74]	1(17)
Positive impact of cancer (e.g., improved relationships, future planning/goals, supportive workplace, outcomes, experiences); PTG	[38, 46, 51]	3(13)	[23, 57, 58]	3(30)	[72]	1(17)
Positive psychological well-being; overall happiness; optimism; vitality; maturity (e.g., inner change; im- or maturity compared to peers); satisfaction (e.g., life, career)	[22, 45, 48, 51, 61]	5(22)	[54]	1(10)	[74]	1(17)
Resilience (e.g., new sense of identity, achieving normalcy, finding meaning in experience, different view of life); positive consequences of surviving	[50, 51, 53]	3(13)				
Religion/spirituality; spiritual well-being; increased faith; relationship with God/church as a primary source of inspiration	[51, 53]	2(9)	[23]	1(10)	[72]	1(17)
Coping (e.g., with challenges of cancer survivorship; with cancer-related work challenges)			[54, 68]	2(20)		
Psychological/emotional needs; need for psychological support; quality of care	[50, 51]	2(9)	[58]	1(10)	[71, 72]	2(33)
Information/service needs; wanting more information about illness; counseling	[22, 51]	2(9)	[23]	1(10)	[72]	1(17)
Spiritual needs/support			[68]	1(10)		
Need for age-specific care; specialized structure programming needs			[58]	1(10)	[71]	1(17)
Unmet needs (e.g., nutritional and insurance support, survivorship concerns, social opportunities, flexible treatment, peer support, guidance for future work-related goals)			[58]	1(10)		
Needed additional support (e.g., educational adjustments, counseling, specialized teaching) for physical/mental changes	[50]	1(4)				

*AYACS* Adolescents and young adult cancer survivors; *CCS* Childhood cancer survivors; *HRQoL* Health-related quality of life; *PTSD* Post-Traumatic Stress Disorder; *PTG* Post-traumatic growth; *PTSS* Post-Traumatic Stress Symptoms; *QoL* Quality of life; Ref.: References

^a^If there was no clear information about the age at diagnosis (but it was clearly not adult cancer) or study population comprises both children and AYAs, the category mixed/unclear was used

^b^Reviews in the overall quality of life section explicitly mentioned QoL or HRQoL and could have had additional discussion of related outcomes as well

#### Mental health and emotional functioning

Outcomes within the domain *mental health and emotional functioning* were reported in both CCS (n = 15, 65%) [22, 38, 41, 42, 44, 45, 49, 51–53, 59, 61, 66, 67] and AYACS reviews (n = 9, 90%) [23, 55, 58, 62–64, 68] (Table 3). Depressive symptoms were reported in seven AYACS reviews (70%)[55, 57, 58, 62–64, 68] compared to only five CCS reviews (22%) [22, 42, 45, 49, 59], and anxiety was reported in both AYACS reviews (n = 4, 40%)[55, 58, 62, 68] and CCS (n = 5, 22%)[22, 42, 59, 61, 66]. Overall distress (n = 4, 40%) [57, 62, 63, 68] and fear of cancer recurrence (n = 4, 40%)[23, 58, 64, 68] were more prominent in AYACS reviews compared to CCS reviews (n = 4, 17% [22, 42, 45, 52], n = 4, 17%[51, 53, 66, 67], respectively). Psychological well-being (n = 4, 17%) [45, 51, 52, 61] and emotional functioning (n = 5, 22%) [22, 44, 45, 52, 53] were more prominent in CCS reviews compared to AYACS reviews (n = 1, 10% [68] and n = 1, 10% [23], respectively). Worries and concerns pertaining to cancer and its treatment (n = 3, 13%) [45, 52, 66], andlife plans (n = 1, 4%) [53] were reported in reviews on CCS, whereas AYACS reviews focused on difficulty coming to terms being neither sick nor healthy (n = 1, 10%) [58].

#### Cognitive functioning

Outcomes relating to *cognitive functioning* were predominantly reported in CCS (n = 8, 35%) [22, 40, 43, 45, 50, 51, 53, 60] compared to AYACS reviews (n = 2, 20%) [57, 68] (Table 3). Cognitive difficulties were reported in five CCS reviews (22%) [22, 45, 50, 51, 60] and only one AYACS review (10%) [68], similar to memory, language, attention, and concentration outcomes which were reported by four CCS reviews (17%) [40, 50, 51, 53] and one AYACS review (10%) [57]. Other outcomes reported by CCS reviews included intellectual functioning (n = 2, 9%) [40, 43], executive functioning (n = 2, 9%) [40, 43], and visuospatial skills (n = 2, 9%) [40, 53].

#### Physical functioning

*Physical functioning* outcomes were reported in both CCS (n = 9, 39%) [22, 42, 44, 45, 50–53, 60, 61] and AYACS (n = 5, 50%) [23, 54, 58, 63, 68] (Table 3). Physical health and Functional status were reported in seven CCS reviews (30%)[22, 44, 45, 50–52, 61] and four AYACS (40%) [23, 58, 63, 68] reviews. Sensory and motor skills were only reported in CCS reviews (n = 4, 17%) [45, 50, 51, 60]. Symptoms such as sleepiness and fatigue were reported in six CCS reviews (26%) [42, 45, 50, 51, 53, 60] compared to only two AYACS reviews (20%) [23, 63]. Yet, other symptoms were only reported in CCS reviews, including pain (n = 3, 13%) [42, 45, 60], loss of appetite and weight loss/gain (n = 1, 4%)[53], vision and speech issues (n = 2, 9%) [45, 51], and treatment discomfort (n = 2, 9%) [45, 52].

#### Social and romantic relationships, and sexual and reproductive health

Outcomes relating to *social and romantic relationships, and sexual and reproductive health* were commonly reported by both CCS (n = 15, 65%) [22, 38, 41, 44, 45, 48–53, 59–61, 66] and AYACS reviews (n = 7, 70%) [23, 56–58, 64, 68] (Table 3). Outcomes pertaining to social relationships, social connection, and communication were reported by four AYACS reviews (40%) [23, 58, 63, 68], and four CCS reviews (17%) [41, 50, 51, 59]. The impact on friendships (CCS: n = 5, 22% [49–51, 53, 59]; AYACS: n = 2, 20% [23, 68]) and family functioning (CCS: n = 4, 17% [41, 44, 53, 60]; AYACS: n = 1, 10% [23]) were reported by both groups. Reports of social challenges, including bullying, feeling different from peers and teasing were slightly more prominent in CCS reviews (n = 6, 26%) [48, 50, 51, 53, 59, 66] compared to AYACS reviews (n = 2, 20%) [57, 68]. Sexual functioning (n = 3, 30%) [56, 58, 68] and fertility-related outcomes (n = 4, 40%) [23, 58, 64, 68] were predominantly reported in AYACS reviews compared to CCS reviews (n = 4, 17% [22, 48, 51, 53] and n = 3, 13% [48, 51, 66], respectively). The desire to have children and family planning was only reported in AYACS reviews (n = 3, 30%) [23, 58, 64].

#### Health behavior

*Health behavior* outcomes were predominantly reported in CCS reviews (n = 11, 48%) [22, 38, 39, 41, 45, 48, 50, 51, 53, 61, 67] compared to AYACS reviews (n = 2, 20%) [58, 68] (Table 3). Both groups reported outcomes pertaining to physical activity (CCS: n = 2, 9% [38, 39]; AYACS: n = 1, 10% [58]), social activities (CCS: n = 4, 17% [22, 48, 50, 51]; AYACS: n = 2, 20% [58, 68]), smoking (CCS: n = 1, 4% [67]; AYACS: n = 2, 20% [58, 68]) and alcohol use (CCS: n = 9% [61, 67]; AYACS: n = 2, 20% [58, 68]). However, eating disorders (n = 1, 4%) [53] and anti-social behaviors (n = 2, 9%) [41, 49] were only reported in CCS reviews, whereas drugs (n = 1, 10%) [68] and diet outcomes were only reported in AYACS reviews (n = 1, 10%) [68].

#### Education, employment, and financial toxicity

Outcomes relating to *education, employment, and financial toxicity* were frequently reported by both CCS reviews (n = 14, 61%) [22, 37, 38, 44, 45, 48–51, 53, 59–61, 66] and AYACS (n = 6, 60%) [23, 54, 57, 58, 63, 68] (Table 3). Education outcomes, including academic achievement and educational pathways, were reported in both groups: CCS (n = 6, 26%) [22, 37, 48, 49, 51, 59] and AYACS (n = 3, 30%) [23, 54, 68]. School functioning (n = 5, 22%) [44, 45, 48, 50, 60] and school re-entry (n = 4, 17%) [38, 48, 50, 66] were more predominantly reported in CCS compared to AYACS reviews (n = 1, 10% [58] and n = 1, 10% [54], respectively). Special education needs (n = 4, 17%) [37, 50, 51, 60] academic delay (n = 4, 17%) [37, 48–50] were only reported in CCS reviews. Work outcomes such as employment after cancer and career changes were reported in AYACS (n = 5, 50%) [23, 54, 57, 58, 68] and CCS reviews (n = 5, 22%) [22, 37, 48, 49, 51]. Most financial outcomes were reported by AYACS reviews compared to CCS reviews, including financial difficulties (AYACS: n = 5, 50% [23, 54, 57, 58, 68]; CCS: n = 1, 4% [51]) and affording housing (AYACS: n = 1, 10% [68]; CCS: n = 1, 4% [51]). The impact of insurance was only reported in AYACS reviews (n = 2, 20%) [54, 68].

#### Self-perception

Outcomes of the *self-perception* domain were reported in both CCS reviews (n = 9, 39%) [38, 48, 51, 53, 59–61, 66] and AYACS reviews (n = 5, 50%) [55, 57, 58, 68] (Table 3). Body image outcomes were reported in six CCS reviews (26%) [48, 51–53, 61, 66] and two AYACS reviews (20%) [57, 58]. Self-image outcomes were reported in two AYACS reviews (20%) [58, 63] and two CCS reviews (9%) [59, 61]. Self-confidence (n = 3, 13%) [38, 61, 66] and self-care (n = 1, 4%) [51] were only reported in CCS reviews. However, identity outcomes (n = 2, 20%) [57, 68] were only reported in AYACS reviews not in CCS.

#### Positive outcomes, coping, and unmet needs

*Positive outcomes, coping, and unmet needs* were reported in both CCS reviews (n = 9, 39%) [22, 38, 45, 46, 48, 50, 51, 53, 61] and AYACS reviews (n = 5, 50%) [23, 54, 57, 58, 68] (Table 3). Overall happiness and optimism were reported in five CCS reviews (22%) [22, 45, 48, 51, 61] compared to only one AYACS review (10%) [54], yet the positive impact of cancer was reported in AYACS reviews (n = 3, 30%) [23, 57, 58] and CCS reviews (n = 3, 13%) [38, 46, 51]. Coping outcomes were only reported in AYACS reviews (n = 2, 20%) [54, 68], and resilience outcomes were only reported in CCS reviews (n = 3, 13%) [50, 51, 53]. Psychological and emotional needs (CCS: n = 2, 9% [50, 51]; AYACS: n = 1, 10% [58]) and information needs (CCS: n = 2, 9% [22, 51]; AYACS: n = 1, 10% [23]) were reported in both groups. However, outcomes including spiritual needs (n = 1, 10%) [68], and need for age-specific care (n = 1, 10%) [58] were only reported in AYACS reviews, but additional support needs such as counseling (n = 1, 4%) [50] were only reported in CCS reviews.

## Discussion and future directions

This umbrella review aimed to map and identify similarities and differences between QoL challenges reported in the literature for both CCS and AYACS. Overall, we included 39 reviews and categorized the QoL outcomes into eight domains affecting CCS and/or AYACS, or a mixture of the two. QoL domains such as *mental health and emotional functioning*, *social and romantic relationships, sexual and reproductive health,* and *education, employment, and financial toxicity* were reported in most reviews across both groups of young survivors. The *cognitive functioning* and *health behavior* domains were most prominent in CCS reviews. Reviews on CCS had a greater focus on outcomes relating to emotional functioning, cognitive difficulties, social challenges (e.g., bullying, peer exclusion), school functioning (e.g., school re-entry), body image and overall happiness, whereas AYACS reviews focused more on depressive symptoms, outcomes related to sexual health and reproductive health (e.g., fertility, sexual functioning, family planning), employment (e.g., work, career), financial difficulties, self-image and identity.

An important observation was that 59% of the 39 reviews included in this umbrella review focused on CCS, compared to only 26% on AYACS, and 15% on mixed/unclear populations. However, the rising number of publications on AYAs over the last two decades signifies their acknowledgment as a vulnerable patient group [12], underscoring their importance in research and practice. Promisingly, with 92% of publications in this review being published in the last 10 years, it suggests an overall increase in interest and attention of QoL challenges in both populations.

The variability in age ranges at diagnosis used in the reviews suggests a lack of shared understanding or awareness of the necessity of delineating between the two populations, as suggested by the four reviews with an unclear CCS/AYACS population. The work by Darlington et al. 2022 was one of the first to begin distinguishing between the age-specific psychosocial requirements of CCS and AYACS [29]. This umbrella review builds upon the conclusion of this work by providing evidence that although some outcomes may be similar across two populations, for example, the importance of physical functioning, others may be different due to different developmental disruptions. For example, although both populations may experience challenges with cognitive functioning, for CCS this may be more impactful due to the delays it may cause to fundamental neurocognitive development (e.g., vocabulary and language comprehension) [75], yet for AYACS it may be more of an issue to its impact on their ability concentrate at work [57].

The high number of reviews reporting an impact of cancer on *mental health and emotional functioning* shows the importance and similarity of these outcomes for young survivors, but they may be driven by different experiences. Although CCS, particularly those diagnosed with cancer at a very young age, may not always remember their lives before cancer [76] and are therefore dependent from the narrative of their family, both populations may have a similar need to process their cancer experience, experiencing cancer-related distress and fear of cancer recurrence. Not only can these overlaps be explained by the proximity of the age groups, but also that these QoL outcomes are ones that stretch across most age-ranges, including adult cancer survivors and elderly cancer survivors [77, 78]. Yet, it is the overlap in QoL outcomes that are most pertinent to the childhood and AYA age-ranges that require more focus. For example, sexual and reproductive health are important to report for both CCS and AYACS, as it has an impact on the rest of their lives irrespective of their diagnosis age. However, it is less likely that CCS have been sexually active or made concrete family planning decisions before their cancer diagnosis, whereas AYACS may need to adjust their sexual functioning or previous family planning after cancer. However, it could be questioned why, in this umbrella review, the desire to have children and family planning was only reported in AYACS reviews, despite the age-range of CCS reaching up to 21 years of age. El Alaoui-Lasmaili et al. 2023 [69] conducted a systematic review in children and AYAs investigating fertility discussions and concerns, therefore it is recognised that it may be the restrictions of this umbrella’s review’s search strategy that limits the number of reviews focusing on this outcome for younger survivors.

Social relationships and support from family and friends are crucial for both age groups and are therefore addressed with similar frequency in reviews of CCS and AYACS. It is important to recognize that whilst many social outcomes will be consistent following treatment and throughout survivorship, they may evolve as the years since treatment completion increase. Often, reviews within this umbrella review only reported age at diagnosis, yet it is age in survivorship that may be a greater indicator of the social challenges they experience. For example, survivors have been found to report growing up faster as a result of their diagnosis, and therefore note the social cost of early maturation and isolation from peers [79]. However, a longer amount of time since treatment completion may result in a diminishing impact of these issues, but perhaps introduce other challenges such as lower life satisfaction in adulthood [80].

In relation to the *education, employment, and financial toxicity* domain, the main differences in outcomes could be explained by the age-differences between CCS and AYACS. Whereas CCS reviews reported outcomes relating to returning to school and the need for academic assistance, AYACS reviews had greater focus on career changes and financial implications of treatment. The lives of CCS change after their return to school environment, especially in terms of building new relationships with peers, attending school due to physical effects such as fatigue, medical care, or parents’ concerns [38, 48, 50, 51, 61, 66]. For some CCS, school re-entry is very stressful, and others report to gain a period of “fame” after returning to school [38, 48, 50, 66] For AYACS, returning to work [23, 54, 57, 58, 68] and stabilizing their financial situations [23, 54, 57, 58, 68] may be vital due to having family to care for and bills to pay. Following treatment completion, CCS and AYACS will be navigating very different life milestones, and they must strive to do this whilst overcoming the long-term effects of their diagnosis.

### In light of our findings, the fundamental question is: does QoL depend on the cancer experience itself, the age at diagnosis, or the current age or stage of life?

In addition to this overarching question, it is imperative to outline future research directions that could be assessed for both CCS and AYACS, since there is a clear need for a better understanding of unique developmental tasks that young people experience:

1. **Unlock similarities and differences in QoL** among CCS and AYACS through qualitative research
2. **Track psychosocial changes longitudinally** to identify patterns of vulnerability and long-term impact of QoL
3. **Focus on transition periods**, such as the shift from childhood to adolescence to young adult follow-up care, to address challenges and opportunities, ensure continuity of care and address age-specific needs
4. **Adopt a life course perspective** to understand the impact of early experiences on long-term outcomes, including education, career, mental health and other QoL domains
5. **Encourage collaborative and interdisciplinary research** involving healthcare providers, psychologists, educators, and researchers to address the multifaceted nature of survivorship issues

By focusing on these future directions, researchers can contribute to a more nuanced and holistic understanding of the survivorship experiences of both CCS and AYACS, leading to improved interventions and support tailored to their unique needs in the future. This umbrella review will inform ongoing work by the EORTC Quality of Life Group to propose a measurement strategy for CCS and AYACS.

### Strengths and limitations

This umbrella review presents a comprehensive examination of QoL outcomes affecting both CCS and AYACS, providing valuable insights into their survivorship experiences. Strengths of the review include its thorough coverage of QoL outcomes and identification of gaps in the existing literature which can guide future research efforts. However, limitations are noted, including a lack of clarity in the definition of “survivors” and variations in the age ranges used across studies, which may affect the interpretation and comparability of findings. Additionally, while the review aims to provide a comprehensive summary of outcomes, there may be a trade-off between comprehensiveness and the loss of detailed information. Moreover, it remains uncertain whether the outcomes identified are a reflection of the focus of the reviews captured rather than the prevalence of QoL concerns. Despite these limitations, the review offers valuable insights into QoL outcomes in CCS and AYACS and underscores the need for clarity and consistency in future research endeavors.

## Conclusion

In conclusion, this umbrella review maps QoL outcomes across eight domains among CCS and AYACS, highlighting shared experiences and challenges that are unique to both groups. QoL domains such as mental health and emotional functioning, social and romantic relationships, sexual and reproductive health, and education, employment, and financial toxicity were the most commonly reported across all reviews. The cognitive functioning and health behavior domains were most prominent in CCS reviews. Reviews on CCS had a greater focus on outcomes relating to emotional functioning, cognitive difficulties, social challenges, and education, whereas AYACS reviews had a greater focus on depressive symptoms, outcomes related to sexual health and reproductive health, employment, and financial difficulties. Future research directions offer a roadmap for developing tailored questionnaires to capture their unique developmental tasks, facilitating a more comprehensive assessment of their QoL, alongside embracing a life course perspective, and exploring emerging technologies to further enhance support.

## Supplementary Information

Below is the link to the electronic supplementary material.

Supplementary file1 (DOCX 138 kb)

## Data Availability

The data that support the findings of this study are available from the corresponding author upon reasonable request.

## Abbreviations

ALL: Acute lymphoid leukemia
AYAs: Adolescents and young adults
AYACS: Adolescent and young adult cancer survivor
BI: Body image
BNI: British nursing index
Cancerlit: Cancer literature
CCS: Childhood cancer survivor
CNS: Central nervous system
Cochrane: The crochrane collaboration
CINAHL: Cumulative index to nursing and allied health literature
EBSCOhost: Electronic business services corporation host
Embase: Excerpta medica database
ERIC: Education resources information center
FIS: FIS education electronic database
HRQoL: Health-related quality of life
IBECS: Spanish bibliographic index of health sciences
LILACS: Latin American and Caribbean health sciences literature
N: Number
PILOTS: Published international literature on traumatic stress
PM: PubMed central
PTSD: Post-traumatic stress disorder
PTG: Post-traumatic growth
QoL: Quality of life
Ref.: Reference
YASCC: Young adult survivors of childhood cancer
