# Supplementary material for: Mapping and comparing the quality of life outcomes in childhood and adolescent and young adult cancer survivors: an umbrella review and future directions
Source: Qual Life Res. 2024 Dec 19;34(3):633–56. doi: 10.1007/s11136-024-03825-7 (PMC11919941; doi:10.1007/s11136-024-03825-7)
Supplement: Supplementary file 1 — Supplementary file1 (DOCX 138 kb) [file 11136_2024_3825_MOESM1_ESM.docx]

**[Quality of Life Research]**

**Appendix to the manuscript**

**Mapping and comparing the quality of life outcomes after cancer as a young person:**

**an umbrella review and future directions**

Céline Bolliger, Kirsty Way, Gisela Michel, Samantha C. Sodergren and Anne-Sophie Darlington^*^

On behalf of the EORTC Quality of Life Group

^*^Corresponding author

**Overview**

**Supplementary Information 1** - PRISMA 2020 Checklist

**Supplementary Information 2** - Search Strategy for PubMed, PsycINFO and CINAHL

**Supplementary Information 3** – AMSTAR, questions

**Supplementary Information 4** – AMSTAR, quality assessment for the included reviews

**Supplementary Information 1 - PRISMA 2020 Checklist**


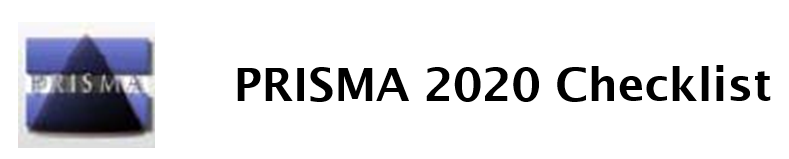


| **Section and Topic** | **Item #** | **Checklist item** | **Location where item is reported** |
| --- | --- | --- | --- |
| **TITLE** | | |  |
| Title | 1 | Identify the report as a systematic review. | Title page |
| **ABSTRACT** | | |  |
| Abstract | 2 | See the PRISMA 2020 for Abstracts checklist. | 2 |
| **INTRODUCTION** | | |  |
| Rationale | 3 | Describe the rationale for the review in the context of existing knowledge. | 4-5 |
| Objectives | 4 | Provide an explicit statement of the objective(s) or question(s) the review addresses. | 5 |
| **METHODS** | | |  |
| Eligibility criteria | 5 | Specify the inclusion and exclusion criteria for the review and how studies were grouped for the syntheses. | 6 |
| Information sources | 6 | Specify all databases, registers, websites, organisations, reference lists and other sources searched or consulted to identify studies. Specify the date when each source was last searched or consulted. | 6 |
| Search strategy | 7 | Present the full search strategies for all databases, registers and websites, including any filters and limits used. | 6, Supplementary Information 2 |
| Selection process | 8 | Specify the methods used to decide whether a study met the inclusion criteria of the review, including how many reviewers screened each record and each report retrieved, whether they worked independently, and if applicable, details of automation tools used in the process. | 6 |
| Data collection process | 9 | Specify the methods used to collect data from reports, including how many reviewers collected data from each report, whether they worked independently, any processes for obtaining or confirming data from study investigators, and if applicable, details of automation tools used in the process. | 6 |
| Data items | 10a | List and define all outcomes for which data were sought. Specify whether all results that were compatible with each outcome domain in each study were sought (e.g. for all measures, time points, analyses), and if not, the methods used to decide which results to collect. | 7 |
|  | 10b | List and define all other variables for which data were sought (e.g. participant and intervention characteristics, funding sources). Describe any assumptions made about any missing or unclear information. | 7 |
| Study risk of bias assessment | 11 | Specify the methods used to assess risk of bias in the included studies, including details of the tool(s) used, how many reviewers assessed each study and whether they worked independently, and if applicable, details of automation tools used in the process. | 7 |
| Effect measures | 12 | Specify for each outcome the effect measure(s) (e.g. risk ratio, mean difference) used in the synthesis or presentation of results. | NA |
| Synthesis methods | 13a | Describe the processes used to decide which studies were eligible for each synthesis (e.g. tabulating the study intervention characteristics and comparing against the planned groups for each synthesis (item #5)). | 7 |
|  | 13b | Describe any methods required to prepare the data for presentation or synthesis, such as handling of missing summary statistics, or data conversions. | 7 |
|  | 13c | Describe any methods used to tabulate or visually display results of individual studies and syntheses. | 7 |
|  | 13d | Describe any methods used to synthesize results and provide a rationale for the choice(s). If meta-analysis was performed, describe the model(s), method(s) to identify the presence and extent of statistical heterogeneity, and software package(s) used. | 7 |
|  | 13e | Describe any methods used to explore possible causes of heterogeneity among study results (e.g. subgroup analysis, meta-regression). | 7 |
|  | 13f | Describe any sensitivity analyses conducted to assess robustness of the synthesized results. | 7 |
| Reporting bias assessment | 14 | Describe any methods used to assess risk of bias due to missing results in a synthesis (arising from reporting biases). | 7 |
| Certainty assessment | 15 | Describe any methods used to assess certainty (or confidence) in the body of evidence for an outcome. | 7 |
| **RESULTS** | | |  |
| Study selection | 16a | Describe the results of the search and selection process, from the number of records identified in the search to the number of studies included in the review, ideally using a flow diagram. | 8 |
|  | 16b | Cite studies that might appear to meet the inclusion criteria, but which were excluded, and explain why they were excluded. | 8 |
| Study characteristics | 17 | Cite each included study and present its characteristics. | 10-15 |
| Risk of bias in studies | 18 | Present assessments of risk of bias for each included study. | 10-15 |
| Results of individual studies | 19 | For all outcomes, present, for each study: (a) summary statistics for each group (where appropriate) and (b) an effect estimate and its precision (e.g. confidence/credible interval), ideally using structured tables or plots. | NA |
| Results of syntheses | 20a | For each synthesis, briefly summarise the characteristics and risk of bias among contributing studies. | 10-15 |
|  | 20b | Present results of all statistical syntheses conducted. If meta-analysis was done, present for each the summary estimate and its precision (e.g. confidence/credible interval) and measures of statistical heterogeneity. If comparing groups, describe the direction of the effect. | NA |
|  | 20c | Present results of all investigations of possible causes of heterogeneity among study results. | 10-15 |
|  | 20d | Present results of all sensitivity analyses conducted to assess the robustness of the synthesized results. | 10-15 |
| Reporting biases | 21 | Present assessments of risk of bias due to missing results (arising from reporting biases) for each synthesis assessed. | 10-15 |
| Certainty of evidence | 22 | Present assessments of certainty (or confidence) in the body of evidence for each outcome assessed. | 10-15 |
| **DISCUSSION** | | |  |
| Discussion | 23a | Provide a general interpretation of the results in the context of other evidence. | 23-25 |
|  | 23b | Discuss any limitations of the evidence included in the review. | 25 |
|  | 23c | Discuss any limitations of the review processes used. | 25 |
|  | 23d | Discuss implications of the results for practice, policy, and future research. | 24 |
| **OTHER INFORMATION** | | |  |
| Registration and protocol | 24a | Provide registration information for the review, including register name and registration number, or state that the review was not registered. | 6 |
|  | 24b | Indicate where the review protocol can be accessed, or state that a protocol was not prepared. | 6 |
|  | 24c | Describe and explain any amendments to information provided at registration or in the protocol. | NA |
| Support | 25 | Describe sources of financial or non-financial support for the review, and the role of the funders or sponsors in the review. | Title page |
| Competing interests | 26 | Declare any competing interests of review authors. | Title page |
| Availability of data, code and other materials | 27 | Report which of the following are publicly available and where they can be found: template data collection forms; data extracted from included studies; data used for all analyses; analytic code; any other materials used in the review. | Title page |

*From:*  Page MJ, McKenzie JE, Bossuyt PM, Boutron I, Hoffmann TC, Mulrow CD, et al. The PRISMA 2020 statement: an updated guideline for reporting systematic reviews. BMJ 2021;372:n71. doi: 10.1136/bmj.n71

For more information, visit: <http://www.prisma-statement.org/>

**Supplementary information 2 - Search Strategy for PubMed, PsycINFO and CINAHL**

| **PubMed Search** | | |
| --- | --- | --- |
| *Search ID** | *Search terms* | *Reference* |
| #1 | "Child"[Mesh] OR "Adolescence"[Mesh] | Developed |
| #2 | child*[Title/Abstract] OR adolescen*[Title/Abstract] OR teen*[Title/Abstract] OR pediatric[Title/Abstract] OR paediatric[Title/Abstract] | adapted from Roser et al. (2019)^1^ |
| #3 | "Adolescent"[Mesh] OR "Young Adult"[Mesh] | Developed |
| #4 | ("young adult"[Title/Abstract] OR "young person*"[Title/Abstract] OR "young people"[Title/Abstract] OR AYA[Title/Abstract] OR TYA[Title/Abstract] OR adolescen*[Title/Abstract] OR teen*[Title/Abstract] OR youngster*[Title/Abstract] OR pre-teen[Title/Abstract]) | adapted from Roser et al.(2019)^1^ |
| #5 | neoplasm*[Title/Abstract] OR cancer*[Title/Abstract] OR carcinoma*[Title/Abstract] OR tumor*[Title/Abstract] OR tumour*[Title/Abstract] OR malignan*[Title/Abstract] OR leukemia*[Title/Abstract] OR leukaemia*[Title/Abstract] OR sarcoma*[Title/Abstract] OR radiotherapy[Title/Abstract] OR chemotherapy[Title/Abstract] | adapted from Roser et al.(2019) ^1^ |
| #6 | "Quality of Life"[Mesh] OR "Personal Autonomy"[Mesh] OR "Social Support"[Mesh] OR "Self Concept"[Mesh] OR "Patient Satisfaction"[Mesh] OR "Religion"[Mesh] OR "Personal Satisfaction"[Mesh] OR "Happiness"[Mesh] OR "Financial Support"[Mesh] OR "Activities of Daily Living"[Mesh] OR "Quality-Adjusted Life Years"[Mesh] OR "Family Relations"[Mesh] | Developed |
| #7 | (“Quality of life”[MAJR] OR “Quality of life”[TIAB] OR “Life quality”[tiab] OR “personal satisfaction”[MAJR] OR “personal satisfaction”[TIAB] OR “patient satisfaction”[MAJR] OR “patient satisfaction”[TIAB] OR “Activities of Daily Living”[MAJR] OR “Activities of Daily Living”[TIAB] OR “Quality-Adjusted Life Years”[MAJR] OR “Quality adjusted life year*”[tiab] OR “Personal autonomy”[MAJR] OR “Personal autonomy”[TIAB] OR “Happiness”[MAJR] OR “Happiness”[TIAB] OR “Patient preference*”[TIAB] OR “fear of death”[TIAB] OR “Self-Concept”[MAJR:NOEXP] OR “Self-concept”[tiab] OR “Family Relations”[MAJR:NOEXP] OR “family relation*”[tiab] OR “Religion”[MAJR:NOEXP] or “Religion”[TIAB] or “social support”[tiab] or “Social Support”[ MAJR] OR “financial support”[tiab] OR “Financial Support”[ MAJR] OR “positive experience”[tiab]) | Adapted from the Biblio 3S (2017)^2^ |
| #8 | ("Systematic Review"[Publication Type:NoExp] OR "Systematic Reviews as Topic"[mesh:noexp] OR (("comprehensive"[TIAB] OR "integrated"[TIAB] OR "integrative"[TIAB] OR "mapping"[TIAB] OR "methodology"[TIAB] OR "narrative"[TIAB] OR "scoping"[TIAB] OR "systematic"[TIAB]) AND ("search"[TIAB] OR "searched"[TIAB] OR "searches"[TIAB] OR "studies"[TIAB]) AND ("cinahl"[TIAB] OR "cochrane"[TIAB] OR "embase"[TIAB] OR "psycinfo"[TIAB] OR "pubmed"[TIAB] OR "medline"[TIAB] OR "scopus"[TIAB] OR "web science"[TIAB] OR "bibliographic review"[TIAB:~1] OR "bibliographic reviews"[TIAB:~1] OR "literature review"[TIAB:~1] OR "literature reviews"[TIAB:~1] OR "literature search"[TIAB:~1] OR "literature searches"[TIAB:~1] OR "narrative review"[TIAB:~1] OR "narrative reviews"[TIAB:~1] OR "qualitative review"[TIAB:~1] OR "qualitative reviews"[TIAB:~1] OR "quantitative review"[TIAB] OR "quantitative reviews"[TIAB])) OR "comprehensive review"[TIAB] OR "comprehensive reviews"[TIAB] OR "comprehensive search"[TIAB] OR "comprehensive searches"[TIAB] OR "critical review"[TIAB] OR "critical reviews" [TIAB] OR (("electronic database"[TIAB:~1] OR "electronic databases"[TIAB:~1] OR "databases searched"[TIAB:~3]) AND (eligibility[tiab] OR excluded[tiab] OR exclusion[tiab] OR included[tiab] OR inclusion[tiab])) OR "evidence assessment"[TIAB] OR "evidence review"[TIAB] OR "exploratory review"[TIAB] OR "framework synthesis"[TIAB] OR "Integrated review"[TIAB] OR "integrated reviews"[TIAB] OR "integrative review"[TIAB:~1] OR "integrative reviews"[TIAB:~1] OR "mapping review"[TIAB:~1] OR "meta-review"[TIAB:~1] OR "meta-synthesis"[TIAB:~1] OR "methodology review"[TIAB:~1] OR ("mixed methods"[TIAB:~0] AND "methods review"[TIAB:~1]) OR ("mixed methods"[TIAB:~0] AND "methods synthesis"[TIAB:~1]) OR "overview reviews"[TIAB:~4] OR ("PRISMA"[TIAB] AND "preferred"[TIAB]) OR "PRISMA-P"[TIAB:~0] OR "prognostic review"[TIAB:~1] OR "psychometric review"[TIAB:~1] OR ("rapid evidence"[TIAB:~0] AND "evidence assessment"[TIAB:~0]) OR "rapid realist"[TIAB:~0] OR "rapid review"[TIAB:~1] OR "rapid reviews"[TIAB:~1] OR "realist review"[TIAB:~1] OR "review of reviews"[TIAB:~1] OR "scoping review"[TIAB:~1] OR "scoping reviews"[TIAB:~1] OR "scoping study"[TIAB:~1] OR ("state art "[TIAB:~2] AND "art review"[TIAB:~1]) OR "systematic evidence map"[TIAB] OR "systematic mapping"[TIAB:~1] OR "systematic literature"[TIAB:~1] OR "systematic Medline"[TIAB:~2] OR "systematic PubMed"[TIAB:~2] OR "systematic review"[TIAB:~1] OR "systematic reviews"[TIAB:~1] OR "systematic search"[TIAB:~1] OR "systematic searches"[TIAB:~1] OR "systematical review"[TIAB:~1] OR "systematical reviews"[TIAB:~1] OR "systematically identified"[TIAB:~1] OR "systematically review"[TIAB:~1] OR "systematically reviewed"[TIAB:~1] OR "umbrella review"[TIAB:~1] OR "umbrella reviews"[TIAB:~1] OR "Cochrane Database Syst Rev"[ta] OR "evid rep technol assess full rep"[Journal] OR "evid rep technol assess summ"[Journal]) | Adapted from VonVille (2023)^3^ |
| #9 | #1 OR #2 |  |
| #10 | #3 OR #4 |  |
| #11 | #6 OR #7 |  |
| #12 | #9 OR #10 |  |
| #13 | #12 AND #11 AND #5 AND #8 |  |

| **PsycINFO** | | |
| --- | --- | --- |
| *Search ID** | *Search terms* | *Reference* |
| S1 | TI ( child* OR adolescen* OR teen* OR pediatric OR paediatric ) OR AB ( child* OR adolescen* OR teen* OR pediatric OR paediatric ) OR KW ( child* OR adolescen* OR teen* OR pediatric OR paediatric ) | adapted from Roser et al.(2019) ^1^ |
| S2 | TI ( (“young adult” OR “young person*” OR “young people” OR AYA OR TYA OR adolescen* OR teen* OR youngster* OR pre-teen) ) OR AB ( (“young adult” OR “young person*” OR “young people” OR AYA OR TYA OR adolescen* OR teen* OR youngster* OR pre-teen) ) OR KW ( (“young adult” OR “young person*” OR “young people” OR AYA OR TYA OR adolescen* OR teen* OR youngster* OR pre-teen) ) | adapted from Roser et al.(2019)^1^ |
| S3 | TI ( neoplasm* OR cancer* OR carcinoma* OR tumor* OR tumour* OR malignan* OR leukemia* OR leukaemia* OR sarcoma* OR radio therapy OR chemotherapy ) OR AB ( neoplasm* OR cancer* OR carcinoma* OR tumor* OR tumour* OR malignan* OR leukemia* OR leukaemia* OR sarcoma* OR radio therapy OR chemotherapy ) OR KW ( neoplasm* OR cancer* OR carcinoma* OR tumor* OR tumour* OR malignan* OR leukemia* OR leukaemia* OR sarcoma* OR radio therapy OR chemotherapy ) | adapted from Roser et al.(2019) ^1^ |
| S4 | DE "Quality of Life" OR DE "Health Related Quality of Life" OR DE "Psychosocial Outcomes" | developed |
| S5 | TI ( ("Quality of life" OR "Life quality" or "Quality adjusted life year*" or "patient satisfaction" OR "Patient preference*" OR "Activities of daily living" OR "Personal autonomy" OR "Happiness" OR "fear of death" OR "Self-concept" or "religion" OR "family relation*" or "social support" or "financial support" OR "positive experience") OR Abstract: ("Quality of life" OR "Life quality" or "Quality adjusted life year*" or "patient satisfaction" OR "Patient preference*" OR "Activities of daily living" OR "Personal autonomy" OR "Happiness" OR "fear of death" OR "Self-concept" or "religion" OR "family relation*" or "social support" or "financial support" OR "positive experience") OR Index Terms: "Quality of life" OR "Client satisfaction" OR "Satisfaction" OR "Life Satisfaction" OR "Activities of Daily Living" OR "Happiness" OR "Self-Concept" OR "Religion" OR "Family relations" OR "Social Support" ) OR AB ( ("Quality of life" OR "Life quality" or "Quality adjusted life year*" or "patient satisfaction" OR "Patient preference*" OR "Activities of daily living" OR "Personal autonomy" OR "Happiness" OR "fear of death" OR "Self-concept" or "religion" OR "family relation*" or "social support" or "financial support" OR "positive experience") OR Abstract: ("Quality of life" OR "Life quality" or "Quality adjusted life year*" or "patient satisfaction" OR "Patient preference*" OR "Activities of daily living" OR "Personal autonomy" OR "Happiness" OR "fear of death" OR "Self-concept" or "religion" OR "family relation*" or "social support" or "financial support" OR "positive experience") OR Index Terms: "Quality of life" OR "Client satisfaction" OR "Satisfaction" OR "Life Satisfaction" OR "Activities of Daily Living" OR "Happiness" OR "Self-Concept" OR "Religion" OR "Family relations" OR "Social Support" ) OR KW ( ("Quality of life" OR "Life quality" or "Quality adjusted life year*" or "patient satisfaction" OR "Patient preference*" OR "Activities of daily living" OR "Personal autonomy" OR "Happiness" OR "fear of death" OR "Self-concept" or "religion" OR "family relation*" or "social support" or "financial support" OR "positive experience") OR Abstract: ("Quality of life" OR "Life quality" or "Quality adjusted life year*" or "patient satisfaction" OR "Patient preference*" OR "Activities of daily living" OR "Personal autonomy" OR "Happiness" OR "fear of death" OR "Self-concept" or "religion" OR "family relation*" or "social support" or "financial support" OR "positive experience") OR Index Terms: "Quality of life" OR "Client satisfaction" OR "Satisfaction" OR "Life Satisfaction" OR "Activities of Daily Living" OR "Happiness" OR "Self-Concept" OR "Religion" OR "Family relations" OR "Social Support" ) | Adapted from the Biblio 3S (2017)^2^ |
| S6 | (DE "Systematic Review") OR (DE "Meta Analysis") | developed |
| S7 | TI ( systematic* N3(review* OR overview*) ) OR AB ( systematic* N3(review* OR overview*) ) OR KW ( systematic* N3(review* OR overview*) | developed |
| S8 | S1 OR S2 |  |
| S9 | S4 OR S5 |  |
| S10 | S6 OR S7 |  |
| S11 | S8 AND S9 AND S10 AND S3 |  |

| **CINAHL** | | |
| --- | --- | --- |
| Search ID* | Search terms | Reference |
| S1 | (MH "Child") OR (MH "Adolescence") | Developed |
| S2 | TI ( child* OR adolescen* OR teen* OR pediatric OR paediatric ) OR AB ( child* OR adolescen* OR teen* OR pediatric OR paediatric ) | adapted from Roser et al. (2019) ^1^ |
| S3 | (MH "Young Adult") OR (MH "Transition to Adulthood") | Developed |
| S4 | TI ( (“young adult” OR “young person*” OR “young people” OR AYA OR TYA OR adolescen* OR teen* OR youngster* OR pre-teen) ) OR AB ( (“young adult” OR “young person*” OR “young people” OR AYA OR TYA OR adolescen* OR teen* OR youngster* OR pre-teen) ) | adapted from Roser et al. (2019) ^1^ |
| S5 | TI ( neoplasm* OR cancer* OR carcinoma* OR tumor* OR tumour* OR malignan* OR leukemia* OR leukaemia* OR sarcoma* OR radio therapy OR chemotherapy ) OR AB ( neoplasm* OR cancer* OR carcinoma* OR tumor* OR tumour* OR malignan* OR leukemia* OR leukaemia* OR sarcoma* OR radio therapy OR chemotherapy ) | adapted from Roser et al. (2019) ^1^ |
| S6 | ("Quality of life" (MM +) OR (TI "Quality of life" OR AB "Quality of life") OR (TI "Life quality" OR AB "Life quality") OR "personal satisfaction" (MM +) OR (TI "personal satisfaction" OR AB "personal satisfaction") OR "patient satisfaction" (MM +) OR (TI "patient satisfaction" OR AB "patient satisfaction") OR "Activities of Daily Living" (MM +) OR (TI "Activities of Daily Living" OR AB "Activities of Daily Living") OR "Quality-Adjusted Life Years" (MM +) OR (TI "Quality adjusted life year*" OR AB "Quality adjusted life year*") OR "Personal autonomy" (MM +) OR (TI "Personal autonomy" OR AB "Personal autonomy") OR (MM Happiness+) OR (TI Happiness OR AB Happiness) OR (TI "Patient preference*" OR AB "Patient preference*") OR (TI "fear of death" OR AB "fear of death") OR (MM Self-Concept) OR (TI Self-concept OR AB Self-concept) OR "Family Relations" (MM ) OR (TI "family relation*" OR AB "family relation*") OR (MM Religion) OR (TI Religion OR AB Religion) OR (TI "social support" OR AB "social support") OR "Social Support[ MAJR]" OR (TI "financial support" OR AB "financial support") OR "Financial Support[ MAJR]" OR (TI "positive experience" OR AB "positive experience")) | Adapted from the Biblio 3S (2017)^2^ |
| S7 | (MH "meta analysis" OR MH "systematic review" OR MH "Technology, Medical/EV" OR PT "systematic review" OR PT "meta analysis" OR (((TI systematic* OR AB systematic*) N3 ((TI review* OR AB review*) OR (TI overview* OR AB overview*))) OR ((TI methodologic* OR AB methodologic*) N3 ((TI review* OR AB review*) OR (TI overview* OR AB overview*)))) OR (((TI quantitative OR AB quantitative) N3 ((TI review* OR AB review*) OR (TI overview* OR AB overview*) OR (TI synthes* OR AB synthes*))) OR ((TI research OR AB research) N3 ((TI integrati* OR AB integrati*) OR (TI overview* OR AB overview*)))) OR (((TI integrative OR AB integrative) N3 ((TI review* OR AB review*) OR (TI overview* OR AB overview*))) OR ((TI collaborative OR AB collaborative) N3 ((TI review* OR AB review*) OR (TI overview* OR AB overview*))) OR ((TI pool* OR AB pool*) N3 (TI analy* OR AB analy*))) OR ((TI "data synthes*" OR AB "data synthes*") OR (TI "data extraction*" OR AB "data extraction*") OR (TI "data abstraction*" OR AB "data abstraction*")) OR ((TI handsearch* OR AB handsearch*) OR (TI "hand search*" OR AB "hand search*")) OR ((TI "mantel haenszel" OR AB "mantel haenszel") OR (TI peto OR AB peto) OR (TI "der simonian" OR AB "der simonian") OR (TI dersimonian OR AB dersimonian) OR (TI "fixed effect*" OR AB "fixed effect*") OR (TI "latin square*" OR AB "latin square*")) OR ((TI "met analy*" OR AB "met analy*") OR (TI metanaly* OR AB metanaly*) OR (TI "technology assessment*" OR AB "technology assessment*") OR (TI HTA OR AB HTA) OR (TI HTAs OR AB HTAs) OR (TI "technology overview*" OR AB "technology overview*") OR (TI "technology appraisal*" OR AB "technology appraisal*")) OR ((TI "meta regression*" OR AB "meta regression*") OR (TI metaregression* OR AB metaregression*)) OR (TI meta-analy* OR TI metaanaly* OR TI "systematic review*" OR TI "biomedical technology assessment*" OR TI "bio-medical technology assessment*" OR AB meta-analy* OR AB metaanaly* OR AB "systematic review*" OR AB "biomedical technology assessment*" OR AB "bio-medical technology assessment*" OR MW meta-analy* OR MW metaanaly* OR MW "systematic review*" OR MW "biomedical technology assessment*" OR MW "bio-medical technology assessment*") OR ((TI medline OR AB medline OR MW medline) OR (TI cochrane OR AB cochrane OR MW cochrane) OR (TI pubmed OR AB pubmed OR MW pubmed) OR (TI medlars OR AB medlars OR MW medlars) OR (TI embase OR AB embase OR MW embase) OR (TI cinahl OR AB cinahl OR MW cinahl)) OR (SO Cochrane OR SO health technology assessment OR SO evidence report) OR ((TI comparative OR AB comparative) N3 ((TI efficacy OR AB efficacy) OR (TI effectiveness OR AB effectiveness))) OR ((TI "outcomes research" OR AB "outcomes research") OR (TI "relative effectiveness" OR AB "relative effectiveness")) OR (((TI indirect OR AB indirect) OR (TI "indirect treatment" OR AB "indirect treatment") OR (TI mixed-treatment OR AB mixed-treatment) OR (TI bayesian OR AB bayesian)) N3 (TI comparison* OR AB comparison*)) OR ((TI multi* OR AB multi*) N3 (TI treatment OR AB treatment) N3 (TI comparison* OR AB comparison*)) OR ((TI mixed OR AB mixed) N3 (TI treatment OR AB treatment) N3 ((TI meta-analy* OR AB meta-analy*) OR (TI metaanaly* OR AB metaanaly*))) OR (TI "umbrella review*" OR AB "umbrella review*") OR ((TI multi* OR AB multi*) N2 (TI paramet* OR AB paramet*) N2 (TI evidence OR AB evidence) N2 (TI synthesis OR AB synthesis)) OR ((TI multiparamet* OR AB multiparamet*) N2 (TI evidence OR AB evidence) N2 (TI synthesis OR AB synthesis)) OR ((TI multi-paramet* OR AB multi-paramet*) N2 (TI evidence OR AB evidence) N2 (TI synthesis OR AB synthesis)) | Adapted from CADTH Search Filters Database (2024) ^4^ |
| S8 | S1 OR S2 |  |
| S9 | S3 OR S4 |  |
| S10 | S8 OR S9 |  |
| S11 | S5 AND S6 AND S7 AND S10 |  |

**Supplementary information 3 – AMSTAR, questions**

| **AMSTAR quality assessment questions**^5^ |
| --- |
| 1. **Was an " a priori" design provided?**   *The research question and inclusion criteria should be established before the conduct of the review.* |
| 1. **Was there duplicate study selection and data extraction?**   *There should be at least two independent data extractors and a consensus procedure for disagreements should be in place.* |
| 1. **Was a comprehensive literature search performed?**   *At least two electronic sources should be searched. The report must include years and databases used (e.g. Central, EMBASE, and MEDLINE). Key words and/or MESH terms must be stated and where feasible the search strategy should be provided. All searches should be supplemented by consulting current contents, reviews, textbooks, specialized registers, or experts in the particular field of study, and by reviewing the references in the studies found.* |
| 1. **Was the status of publication (i.e. grey literature) used as an inclusion criterion?**   *The authors should state that they searched for reports regardless of their publication type. The authors should state whether or not they excluded any reports (from the systematic review), based on their publication status, language etc.* |
| 1. **Was a list of studies (included and excluded) provided?**   A list of included and excluded studies should be provided |
| 1. **Were the characteristics of the included studies provided?**   *In an aggregated form such as a table, data from the original studies should be provided on the participants, interventions and outcomes. The ranges of characteristics in all the studies analyzed e.g. age, race, sex, relevant socioeconomic data, disease status, duration, severity, or other diseases should be reported.* |
| 1. **Was the scientific quality of the included studies assessed and documented?**   *'A priori' methods of assessment should be provided (e.g., for effectiveness studies if the author(s) chose to include only randomized, double-blind, placebo controlled studies, or allocation concealment as inclusion criteria); for other types of studies, alternative items will be relevant.* |
| 1. **Was the scientific quality of the included studies used appropriately in formulating conclusions?**   *The results of the methodological rigor and scientific quality should be considered in the analysis and the conclusions of the review, and explicitly stated in formulating recommendations.* |
| 1. **Were the methods used to combine the findings of studies appropriate?**   *For the pooled results, a test should be done to ensure the studies were combinable, to assess their homogeneity (i.e. Chisquared test for homogeneity, I2). If heterogeneity exists a random effects model should be used and/or the clinical appropriateness of combining should be taken into consideration (i.e. is it sensible to combine?).* |
| 1. **Was the likelihood of publication bias assessed?**   *An assessment of publication bias should include a combination of graphical aids (e.g., funnel plot, other available tests) and/or statistical tests (e.g., Egger regression test)* |
| 1. **Was the conflict of interest stated?**   *Potential sources of support should be clearly acknowledged in both the systematic review and the included studies.* |

**Supplementary Information 4 – AMSTAR, quality assessment of the included reviews**

|  | **AMSTAR scores** | | | | | |
| --- | --- | --- | --- | --- | --- | --- |
| **Authors (Year)^Ref^** |  | Total n of YES (%) by rater 1 |  | Total n of YES (%) by rater 2 |  | Mean  (rater 1, rater 2) |
| Deegan et al. (2023)^6^ |  | 6 (55) |  | 6 (55) |  | 6 |
| Larsen et al. (2023)^7^ |  | 6 (55) |  | 5 (45) |  | 6 |
| Kappelmann et al. (2023)^8^ |  | 7 (64) |  | 6 (55) |  | 7 |
| Sciancalepore et al. (2023)^9^ |  | 7 (64) |  | 6 (55) |  | 7 |
| Moascato et al. (2022)^10^ |  | 7 (64) |  | 5 (45) |  | 6 |
| Martinez-Santos et al. (2021)^11^ |  | 8 (73) |  | 6 (55) |  | 7 |
| Pahl et al. (2021)^12^ |  | 6 (55) |  | 5 (45) |  | 6 |
| Schulte et al. (2021)^13^ |  | 7 (64) |  | 6 (55) |  | 7 |
| Godoy et al. (2020)^14^ |  | 8 (73) |  | 6 (55) |  | 7 |
| Frederiksen et al. (2019)^15^ |  | 8 (73) |  | 8 (73) |  | 8 |
| Garas et al. (2019)^16^ |  | 8 (73) |  | 6 (55) |  | 7 |
| Nicklin et al. (2019)^17^ |  | 6 (55) |  | 5 (45) |  | 6 |
| Vetsch et al. (2018)^18^ |  | 7 (64) |  | 6 (55) |  | 7 |
| Turner et al. (2018)^19^ |  | 8 (73) |  | 3 (27) |  | 9 |
| McDonell et al. (2017)^20^ |  | 6 (55) |  | 5 (45) |  | 6 |
| Schulte et al. (2017)^21^ |  | 9 (82) |  | 9 (82) |  | 9 |
| Macartney et al. (2014)^22^ |  | 4 (36) |  | 5 (45) |  | 5 |
| Pini et al. (2012)^23^ |  | 6 (55) |  | 4 (36) |  | 5 |
| Klassen et al. (2011)^24^ |  | 5 (45) |  | 4 (36) |  | 5 |
| Nightingale et al. (2011)^25^ |  | 4 (36) |  | 3 (27) |  | 4 |
| Lund et al. (2011)^26^ |  | 4 (36) |  | 4 (36) |  | 4 |
| Clarke et al. (2007)^27^ |  | 6 (55) |  | 3 (27) |  | 5 |
| Eiser et al. (2000)^28^ |  | 4 (36) |  | 3 (27) |  | 4 |
| Altherr et al. (2023)^29^ |  | 6 (55) |  | 6 (55) |  | 6 |
| Tanner et al. (2023)^30^ |  | 5 (45) |  | 4 (36) |  | 5 |
| Osmani et al. (2023)^31^ |  | 9 (82) |  | 9 (82) |  | 9 |
| Bradford et al. (2022)^32^ |  | 6 (55) |  | 6 (55) |  | 6 |
| Stanton et al. (2018)^33^ |  | 6 (55) |  | 7 (64) |  | 7 |
| Stone et al. (2017)^34^ |  | 6 (55) |  | 5 (45) |  | 6 |
| Schilstra et al. (2021)^35^ |  | 7 (64) |  | 8 (73) |  | 8 |
| Barnett et al. (2016)^36^ |  | 6 (55) |  | 7 (64) |  | 7 |
| Quinn et al. (2015)^37^ |  | 6 (55) |  | 4 (36) |  | 5 |
| Gonçalves et al. (2014)^38^ |  | 5 (45) |  | 4 (36) |  | 5 |
| El Alaoui-Lasmali et al. (2023)^39^ |  | 7 (64) |  | 5 (45) |  | 6 |
| Logan et al. (2019)^40^ |  | 7 (64) |  | 5 (45) |  | 6 |
| Galan et al. (2018)^41^ |  | 7 (64) |  | 8 (73) |  | 8 |
| Ismail et al. (2018)^42^ |  | 6 (55) |  | 4 (36) |  | 5 |
| Olson et al. (2015)^43^ |  | 4 (36) |  | 4 (36) |  | 4 |
| Fan et al. (2009)^44^ |  | 5 (45) |  | 3 (27) |  | 4 |

Abbreviations: n: Number; Ref.: Reference

**Cohen’s kappa**: 0.73 (substantial agreement between rater 1 and rater 2).^45^

**References**

1. Altherr A, Bolliger C, Kaufmann M, Dyntar D, Scheinemann K, Michel G, et al. Education, Employment, and Financial Outcomes in Adolescent and Young Adult Cancer Survivors-A Systematic Review. Curr Oncol Tor Ont. 2023 Sep 25;30(10):8720–62.

2. Canadian Health Libraries Association. Qualité de vie [Biblio3S] [Internet]. 2017 [cited 2024 Feb 6]. Available from: https://extranet.santecom.qc.ca/wiki/!biblio3s/doku.php?id=concepts:qualite_de_vie

3. VonVille H. Filters for systematic review, related reviews and meta-analyses [Internet]. 2023 [cited 2024 Feb 6]. Available from: https://www.yopl.info/post/pubmed-research-methodology-search-filters-and-a-couple-of-nifty-limits

4. CADTH. SR / MA / HTA / ITC - CINAHL. [Internet]. CADTH Search Filters Database; 2024 [cited 2014 Feb 6]. Available from: https://searchfilters.cadth.ca/link/98

5. Shea BJ, Grimshaw JM, Wells GA, Boers M, Andersson N, Hamel C, et al. Development of AMSTAR: a measurement tool to assess the methodological quality of systematic reviews. BMC Med Res Methodol. 2007 Feb 15;7:10.

6. Deegan A, Brennan C, Gallagher P, Lambert V, Dunne S. Social support and childhood cancer survivors: A systematic review (2006-2022). Psychooncology. 2023;32(6):819–33.

7. Larsen PA, Amidi A, Ghith N, Winther JF, Pedersen C. Quality of life of adolescent and adult survivors of childhood cancer in Europe—A systematic review. Int J Cancer. 2023 Oct;153(7):1356–75.

8. Kappelmann L, Götte M, Krombholz A, Hüter J, Fischer B. Factors That Influence Physical Activity Behavior in Children and Adolescents During and After Cancer Treatment: A Qualitative Systematic Review of the Literature. Pediatr Exerc Sci. 2023 Oct 27;1–9.

9. Sciancalepore F, Fabozzi F, Albino G, Del Baldo G, Di Ruscio V, Laus B, et al. Frequency and characterization of cognitive impairments in patients diagnosed with paediatric central nervous system tumours: a systematic review. Front Oncol. 2023;13:1198521.

10. Moscato E, Patronick J, Wade SL. Family functioning and adaptation following pediatric brain tumor: A systematic review. Pediatr Blood Cancer. 2022;69(2):e29470.

11. Martinez-Santos AE, Fernandez-De-La-Iglesia JDC, Sheaf G, Coyne I. A systematic review of the educational experiences and needs of children with cancer returning to school. J Adv Nurs. 2021 Jul;77(7):2971–94.

12. Pahl DA, Wieder MS, Steinberg DM. Social isolation and connection in adolescents with cancer and survivors of childhood cancer: A systematic review. J Adolesc. 2021 Feb;87:15–27.

13. Schulte FSM, Patton M, Alberts NM, Kunin-Batson A, Olson-Bullis BA, Forbes C, et al. Pain in long-term survivors of childhood cancer: A systematic review of the current state of knowledge and a call to action from the Children’s Oncology Group. Cancer. 2021 Jan 1;127(1):35–44.

14. Godoy PBG. Assessment of Executive Functions after Treatment of Childhood Acute Lymphoid Leukemia: a Systematic Review. Neuropsychol Rev. 2020;

15. Frederiksen LE, Mader L, Feychting M, Mogensen H, Madanat‐Harjuoja L, Malila N, et al. Surviving childhood cancer: a systematic review of studies on risk and determinants of adverse socioeconomic outcomes. Int J Cancer. 2019 Apr 15;144(8):1796–823.

16. Garas A, McLean LA, De Luca CR, Downie P, McCarthy MC. Health-related quality of life in paediatric patients up to five years post-treatment completion for acute lymphoblastic leukaemia: a systematic review. Support Care Cancer Off J Multinatl Assoc Support Care Cancer. 2019 Nov;27(11):4341–51.

17. Nicklin E, Velikova G, Hulme C, Rodriguez Lopez R, Glaser A, Kwok-Williams M, et al. Long-term issues and supportive care needs of adolescent and young adult childhood brain tumour survivors and their caregivers: A systematic review. Psychooncology. 2019;28(3):477–87.

18. Vetsch J, Wakefield CE, Robertson EG, Trahair TN, Mateos MK, Grootenhuis M, et al. Health-related quality of life of survivors of childhood acute lymphoblastic leukemia: a systematic review. Qual Life Res. 2018 Jun;27(6):1431–43.

19. Turner JK, Hutchinson A, Wilson C. Correlates of post-traumatic growth following childhood and adolescent cancer: A systematic review and meta-analysis. Psychooncology. 2018 Apr;27(4):1100–9.

20. McDonnell GA, Salley CG, Barnett M, DeRosa AP, Werk RS, Hourani A, et al. Anxiety Among Adolescent Survivors of Pediatric Cancer. J Adolesc Health. 2017 Oct;61(4):409–23.

21. Schulte F, Russell KB, Cullen P, Embry L, Fay-McClymont T, Johnston D, et al. Systematic review and meta-analysis of health-related quality of life in pediatric CNS tumor survivors. Pediatr Blood Cancer. 2017 Aug;64(8).

22. Macartney G, Harrison MB, VanDenKerkhof E, Stacey D, McCarthy P. Quality of life and symptoms in pediatric brain tumor survivors: a systematic review. J Pediatr Oncol Nurs Off J Assoc Pediatr Oncol Nurses. 2014 Apr;31(2):65–77.

23. Pini S, Hugh‐Jones S, Gardner PH. What effect does a cancer diagnosis have on the educational engagement and school life of teenagers? A systematic review. Psychooncology. 2012 Jul;21(7):685–94.

24. Klassen AF, Anthony SJ, Khan A, Sung L, Klaassen R. Identifying determinants of quality of life of children with cancer and childhood cancer survivors: a systematic review. Support Care Cancer Off J Multinatl Assoc Support Care Cancer. 2011 Sep;19(9):1275–87.

25. Nightingale CL, Quinn GP, Shenkman EA, Curbow BA, Zebrack BJ, Krull KR, et al. Health-Related Quality of Life of Young Adult Survivors of Childhood Cancer: A Review of Qualitative Studies. J Adolesc Young Adult Oncol. 2011 Sep;1(3):124–32.

26. Lund LW, Schmiegelow K, Rechnitzer C, Johansen C. A systematic review of studies on psychosocial late effects of childhood cancer: structures of society and methodological pitfalls may challenge the conclusions. Pediatr Blood Cancer. 2011 Apr;56(4):532–43.

27. Clarke SA, Eiser C. Health behaviours in childhood cancer survivors: A systematic review. Eur J Cancer. 2007 Jun;43(9):1373–84.

28. Eiser C. Examining the Psychological Consequences of Surviving Childhood Cancer: Systematic Review as a Research Method in Pediatric Psychology. J Pediatr Psychol. 2000 Sep 1;25(6):449–60.

29. Altherr A, Bolliger C, Kaufmann M, Dyntar D, Scheinemann K, Michel G, et al. Education, Employment, and Financial Outcomes in Adolescent and Young Adult Cancer Survivors-A Systematic Review. Curr Oncol Tor Ont. 2023;30(10):8720–62.

30. Tanner S, Engstrom T, Lee WR, Forbes C, Walker R, Bradford N, et al. Mental health patient-reported outcomes among adolescents and young adult cancer survivors: A systematic review. Cancer Med. 2023 Sep;12(17):18381–93.

31. Osmani V, Hörner L, Klug SJ, Tanaka LF. Prevalence and risk of psychological distress, anxiety and depression in adolescent and young adult (AYA) cancer survivors: A systematic review and meta-analysis. Cancer Med. 2023 Sep;12(17):18354–67.

32. Bradford NK, McDonald FEJ, Bibby H, Kok C, Patterson P. Psychological, functional and social outcomes in adolescent and young adult cancer survivors over time: A systematic review of longitudinal studies. Psychooncology. 2022 Sep;31(9):1448–58.

33. Stanton AM, Handy AB, Meston CM. Sexual function in adolescents and young adults diagnosed with cancer: A systematic review. J Cancer Surviv. 2018 Feb;12(1):47–63.

34. Stone DS, Ganz PA, Pavlish C, Robbins WA. Young adult cancer survivors and work: a systematic review. J Cancer Surviv Res Pract. 2017 Dec;11(6):765–81.

35. Schilstra CE, Fardell JE, Burns MA, Ellis SJ, Anazodo AC, Trahair TN, et al. Determinants of social functioning among adolescents and young adults with cancer: A systematic review. Psychooncology. 2021 Oct;30(10):1626–42.

36. Barnett M, McDonnell G, DeRosa A, Schuler T, Philip E, Peterson L, et al. Psychosocial outcomes and interventions among cancer survivors diagnosed during adolescence and young adulthood (AYA): a systematic review. J Cancer Surviv. 2016/02/28 ed. 2016 Oct;10(5):814–31.

37. Quinn GP, Goncalves V, Sehovic I, Bowman ML, Reed DR. Quality of life in adolescent and young adult cancer patients: a systematic review of the literature. Patient Relat Outcome Meas. 2015/03/04 ed. 2015;6:19–51.

38. Gonçalves V, Sehovic I, Quinn G. Childbearing attitudes and decisions of young breast cancer survivors: a systematic review. Hum Reprod Update. 2014 Apr;20(2):279–92.

39. El Alaoui-Lasmaili K, Nguyen-Thi PL, Demogeot N, Lighezzolo-Alnot J, Gross MJ, Mansuy L, et al. Fertility discussions and concerns in childhood cancer survivors, a systematic review for updated practice. Cancer Med. 2023;12(5):6023–39.

40. Logan S, Perz J, Ussher JM, Peate M, Anazodo A. Systematic review of fertility-related psychological distress in cancer patients: Informing on an improved model of care. Psychooncology. 2019;28(1):22–30.

41. Galán S, De La Vega R, Miró J. Needs of adolescents and young adults after cancer treatment: a systematic review. Eur J Cancer Care (Engl). 2018 Nov;27(6):e12558.

42. Ismail Y, Hendry J. Support needs of adolescents’ post-cancer treatment: A systematic review. Radiogr Lond Engl 1995. 2018 May;24(2):175–83.

43. Olson K, Amari A. Self-reported Pain in Adolescents With Leukemia or a Brain Tumor: A Systematic Review. Cancer Nurs. 2015 Oct;38(5):E43-53.

44. Fan SY, Eiser C. Body image of children and adolescents with cancer: A systematic review. Body Image. 2009 Sep;6(4):247–56.

45. Landis JR, Koch GG. The measurement of observer agreement for categorical data. Biometrics. 1977 Mar;33(1):159–74.
